# Deep active learning for suggestive segmentation of biomedical image stacks via optimisation of Dice scores and traced boundary length

**DOI:** 10.1016/j.media.2022.102549

**Published:** 2022-07-24

**Authors:** Alessia Atzeni, Loic Peter, Eleanor Robinson, Emily Blackburn, Juri Althonayan, Daniel C. Alexander, Juan Eugenio Iglesias

**Affiliations:** a Centre for Medical Image Computing, Department of Medical Physics and Biomedical Engineering, University College, London, UK; b Martinos Center for Biomedical Imaging, Massachusetts General Hospital and Harvard Medical School, Boston, USA; c Computer Science and Artificial Intelligence Laboratory, Massachusetts Institute of Technology, Boston, USA

**Keywords:** Segmentation, Deep learning, Active learning, Partial annotation, Histology

## Abstract

Manual segmentation of stacks of 2D biomedical images (e.g., histology) is a time-consuming task which can be sped up with semi-automated techniques. In this article, we present a suggestive deep active learning framework that seeks to minimise the annotation effort required to achieve a certain level of accuracy when labelling such a stack. The framework suggests, at every iteration, a specific region of interest (ROI) in one of the images for manual delineation. Using a deep segmentation neural network and a mixed cross-entropy loss function, we propose a principled strategy to estimate class probabilities for the whole stack, conditioned on heterogeneous partial segmentations of the 2D images, as well as on weak supervision in the form of image indices that bound each ROI. Using the estimated probabilities, we propose a novel active learning criterion based on predictions for the estimated segmentation performance and delineation effort, measured with average Dice scores and total delineated boundary length, respectively, rather than common surrogates such as entropy. The query strategy suggests the ROI that is expected to maximise the ratio between performance and effort, while considering the adjacency of structures that may have already been labelled - which decrease the length of the boundary to trace. We provide quantitative results on synthetically deformed MRI scans and real histological data, showing that our framework can reduce labelling effort by up to 60 – 70% without compromising accuracy.

## 1. Introduction

### 1.1. Motivation

Histology concerns the study of the microanatomy of cells and biological tissue through the microscopic examination of *in vivo* (via biopsy) or *ex vivo* specimens (Culling, 2013). In the case of *ex vivo*, the specimen often consists of a block of tissue embedded in paraffin wax which is cut into thin sections with a microtome, mounted on glass slides, and stained to enhance the visualisation of different microanatomical features (e.g, cytoarchitecture, Bancroft and Gamble 2008). Examples of structural stains commonly used in histology include haematoxylin and eosin (H&E) or Luxol fast blue with cresyl violet (LFB/CV).

While the most common application of human histology is clinical pathology, there is growing interest in its application to 3D atlas building in neuroimaging: since histological sections can be digitised at sub-micron resolution, they enable differentiation and characterisation of structures that are not visible with mm-scale imaging (e.g., MRI). Three-dimensional histological atlases of the brain provide superior levels of detail than their MRI counterparts. Examples of histological atlases in human neuroimaging include Iglesias et al. 2018 and Krauth et al. 2010 (thalamus); Yelnik et al. 2007 (basal ganglia); Chakravarty et al. 2006 (basal ganglia and thalamus); Adler et al. 2014 (hippocampus); Cartmell et al. 2019 (nucleus accumbens); or Amunts et al. 2013 and Ding et al. 2016 (whole human brain).

Building histological atlases presents two major challenges: 3D registration (“histology reconstruction”) and manual delineation. Histology reconstruction is needed because specimen preparation greatly distorts the geometry of the tissue, due to cutting and mounting. A number of dedicated registration algorithms exist to solve this problem (Pichat et al., 2018), many of which rely on an external reference volume acquired prior to cutting (e.g., an MRI scan) in order to avoid errors like “banana effect” (Yang et al., 2012) and z-shift (Pichat et al., 2017).

The second challenge, which we address in this paper, is the need for manually segmenting a large number of thin sections at a very high level of detail, making this task very challenging compared to its mm-scale counterpart. Manual delineation by an expert is considered the gold standard in segmentation, as it is assumed to provide the partitioning closest to the underlying “true” anatomy. However, it is very tedious and time-consuming, which limits sample sizes. This problem is exacerbated in histological atlas building, due to the large number of images that need to be labelled. Moreover, naïve manual segmentation of such datasets is intrinsically inefficient, due to the high similarity between adjacent sections or images in the stack, making a large part of the annotations redundant.

### 1.2. Related work

Semi-automated, interactive, and suggestive (active learning) strategies have been developed to decrease the time required by manual segmentation. Here we provide a short survey of existing techniques under this classification – bearing in mind that there often exists overlap between the groups, e.g., some suggestive methods are interactive, whereas others ultimately seek to build an automated system.

#### 1.2.1. Semi-automated and interactive segmentation

In semi-automated algorithms, the user provides a relatively small amount of input, e.g., a brushstroke, bounding box or landmarks on a single 2D image; or dense labels for one 2D image in a stack (or a slice in a 3D volume). Then, an automated algorithm uses this input to produce a dense segmentation. If these algorithms run quickly enough, they can also be used in an interactive fashion, with the option of progressively refining the segmentation by providing additional input.

One of the first interactive algorithms developed to quickly contour objects within 2D images is *Intelligent Scissors* (Mortensen and Barrett, 1998). In this method, the user is required to interactively place landmark points in proximity to an object edge, after which a minimum cost path to any other point in the image is computed and a live-wire which wraps around the object of interest is generated to ultimately form a closed contour. Another type of interaction consists of drawing a bounding box around the ROI. One representative example of this approach is *GrabCut* (Rother et al., 2004), which treats the pixels outside the bounding box as certain background, and solves the segmentation task by iteratively minimising an energy functional with graph cuts (Boykov et al., 2001) – with possible refinement through additional scribbles. A third type of interaction consists of brushstrokes drawn by the user on the background and foreground. Widespread methods which made use of this type of interaction include *Random Walker* (Grady, 2006) and *GeoS* (Criminisi et al., 2008). In Random Walker, each pixel is assigned the label with the maximum probability of being reached by a random walk starting from the scribbles corresponding to that label. GeoS casts segmentation as approximate energy minimisation problem in a conditional random field, and adds a parallel filter operator built upon geodesic distance maps derived from the scribbles to produce a spatially smooth and contrast-sensitive segmentation.

The aforementioned interactive segmentation algorithms can be either directly applied to 3D volumes (GrabCut, Random Walker, GeoS), or generalised to 3D datasets (see Falcão and Udupa 2000; Grady 2008; Iglesias 2017 for extensions of smart scissors to 3D). However, these 3D versions assume continuous volumes and are not design to cope with stacks of 2D images, as in histology.

A different approach for semi-automated segmentation, which has proven effective with 2D images, 3D volumes, and stacks of images, is to use a supervised approach, using partial manual labels as training data. One family of such approaches is registration-based segmentation, best represented by its multi-atlas version (MAS) (Iglesias and Sabuncu, 2015; Rohlfing et al., 2004). These algorithms non-rigidly register a set of labelled images (“atlases”) to a test image, and merge the deformed labels into a segmentation with a label fusion approach. MAS is directly applicable to stacks of 2D images if the 2D slices used as atlases are fully labelled, since the registration of adjacent images is often accurate (Atzeni et al., 2018).

Another family of supervised techniques which achieves state-of-the-art performance in many automated medical image segmentation tasks relies on voxel classifiers trained on a subset of labelled voxels, e.g., brushstrokes, or labelled slices. This approach is well represented by Arganda-Carreras et al. (2017), included in the Fiji software (Schindelin et al., 2012), which uses brushstrokes to train classical classifiers, such as random forests (Breiman, 2001) and support vector machines (Boser et al., 1992; Cortes and Vapnik, 1995). Such classifiers can be trained and updated quickly, allowing their use in an interactive manner.

Modern approaches rely on convolutional neural networks (CNNs) for segmentation, such as the widespread U-net or V-net architectures (Ronneberger et al., 2015; Milletari et al., 2016). Çiçek et al. (2016) presented a semi-automated setup, in which a deep network is trained from scratch using a sparse set of manual annotations on a volume (e.g., three orthogonal slices), and then applied to the whole volume to obtain a dense segmentation. Training from scratch takes a long time and precludes interactive segmentation. Instead, fine-tuning a pre-trained network can keep the algorithm interactive. For example, Wang et al. (2018) integrates CNNs with bounding box and scribble-based interactions for interactive 2D and 3D medical image segmentation. In the context of natural image segmentation, Jang and Kim (2019); Kontogianni et al. (2020) use the user-provided clicks to finetune the weights of the CNN. In our previous work (Atzeni et al., 2018), we start from a subset of labelled 2D histology sections and produce a dense segmentation of the whole image stack by integrating MAS and a CNN.

A number of modern machine learning methods use “guidance maps” to produce segmentations from an input image and a small number of clicks. These are heat maps which play the role of spatial priors, and are typically fed as an additional input channel to a segmentation CNN. For example, Zhang et al. (2020b) build guidance maps by centring 2D Gaussians on the centre and corners of the object, provided by the user with three clicks. Further clicks can be interactively added at test time to refine the guidance maps. Maninis et al. (2018) is very similar, but allows the user to click on extreme points, determining the location of the Gaussians on the guidance maps. Rather than using Gaussians, Luo et al. (2021) utilise the geodesic distance transform from the user provided clicks (which can be easily updated with additional clicks) to recompute the guidance map and interactively update the segmentation. Majumder and Yao (2019) use multiple, more sophisticated guidance maps which align with both low-level and high-level image structures present in the scene; the former are represented with superpixels, and the latter with region-based object proposals.

Rather than using CNNs to make prediction at the pixel or voxel level, some approaches seek to model the contour of the objects instead. For example, Castrejon et al. (2017) and Acuna et al. (2018) propose interactive methods based on recurrent neural networks that predict the vertices of a polygon outlining the object; this approach enables the user to correct vertices if needed. An evolution of this method (Ling et al., 2019) uses graph convolutions to predict the whole outline at once, rather than sequentially. This idea of fitting contours to has also been applied to medical images by Tian et al. 2020 (prostate segmentation in MRI) and by Williams et al. 2021 (levator hiatus segmentation in ultrasound); the former use a graph CNN, whereas the latter explicitly deform an active contour in order to minimise an energy function combining the output of a (fixed) CNN and the location of landmarks placed by the user.

Rather than assuming that user interactions are independent, some works have sought to exploit their spatial and temporal correlation. For example, Liao et al. (2020) and Ma et al. (2020) model the dynamic process for successive interactions using multi-agent reinforcement learning, where every voxel is an agent. Similarly, Lin et al. (2020) consider the different importance of clicks depending on their order, and propose a “First Click Attention Network” to make better use of the crucial first click. However, none of the aforementioned supervised approaches take into account the contribution of the manual annotations towards the segmentation accuracy in relation to the labelling effort. Such effort can be minimised with active learning frameworks, which automatically suggest which unlabelled images (or regions) to annotate, in order to increase segmentation performance with minimal manual tracing effort, thus allocating human effort more efficiently (Settles, 2012).

#### 1.2.2. Suggestive segmentation with active learning

Active learning is motivated by machine learning problems where data may be abundant but labels are scarce or expensive to obtain. Active learning aims to train a robust supervised or semi-supervised system with as little manual labelling as possible, by suggesting to the user what subset of the available data to label, in order to maximise the performance of the model. In segmentation, if the learning algorithm is fast enough, it can be used in an interactive setting, e.g., requesting the user to manually segment the optimal slice (or slices) in a 3D scan that will enable automated segmentation of the rest of the volume.

Most active learning approaches rely on: *(i)* training an initial classifier with a small set of manually labelled data; *(ii)* estimating the informativeness of unlabelled instances and requesting a label for the most informative sample (e.g., a point, region, image, or volume); *(iii)* retraining the classifier; and *(iv)* iterating *(i)*-*(iii)* until the desired performance level is reached. Common querying (informativeness) criteria for step *(ii)* include: uncertainty sampling, where the active learner selects the instances about which it is least certain how to label (e.g., with maximum entropy, Lewis and Gale 1994); decision-theory, where the framework selects the instance that would lead to the greatest change to the current model if its label was known (e.g., Cohn et al. 1996); or hypothesis space search, which aims to reduce the set of all classification hypotheses consistent with the ground truth (e.g., with query by committee, Freund et al. 1997).

Active learning strategies have been extensively used in computer vision, and have been shown to reduce labelling effort and increase learning speed in object recognition tasks (Aghdam et al., 2019; Kapoor et al., 2007; Vijayanarasimhan and Grauman, 2011), classification problems (Li and Guo, 2013; Wang et al., 2016), and semantic segmentation (Konyushkova et al., 2015; Mackowiak et al., 2018; Siddiqui et al., 2020; Vezhnevets et al., 2012). In the context of medical images, they have been shown to reduce training sample sizes in a wide array of scenarios, both with classical and modern machine learning techniques. Examples of the former include: computer-aided diagnosis of diabetic retinopathy (Sánchez et al. 2010, based on uncertainty sampling and query-by-bagging); segmentation of different organs in CT and MRI scans (Iglesias et al. 2011; Top et al. 2011); or patient-specific 3D heart models for surgical planning (Pace et al. 2015).

The more recent literature on active learning in biomedical imaging relies almost exclusively in deep learning architectures. The type of interaction and strategies to measure the usefulness of a potential annotation vary widely across studies. For example, Yang et al. (2017) assess uncertainty as the variance over bootstrapped predictions (Efron and Tibshirani, 1994) in histology segmentation task, and then use cosine similarity to pick representative examples that are given to the user for manual segmentation. Similarly, Kuo et al. (2018) use an ensemble to estimate uncertainty in an intracranial haemorrhage segmentation task (with the Jensen-Shannon divergence), but also attempt to predict the manual delineation time with a log-linear model; they select for manual segmentation the examples that maximise the sum of uncertainties within a given time budget. In the context of cell instance segmentation, Wang and Yin (2021) also use model ensembles to prompt the user to label the cells that maximise a combination of uncertainty, diversity (measured with a clustering algorithm), and representativeness – measured with the cosine similarity of features extracted with the first 10 layers of VGG16 (Simonyan and Zisserman, 2014) pretrained on ImageNet (Deng et al., 2009).

Rather than using ensembles, Wang et al. (2020) use grouped convolutions to obtain multiple candidate segmentations of fetal brain in MRI, compute a slice-wise uncertainty, and prompts the user to manually correct the slice with highest uncertainty. In a pulmonary nodule detection task, Liu et al. (2020) also avoid ensembles by ranking the samples to be labelled with an uncertainty loss explicitly predicted by multi-layer feature maps. In a vessel segmentation task in ocular images, Xu et al. (2021) choose patches for the user to densely segment by picking the one with the smallest sum of squared deviations from p=0.5, which is a simple surrogate for uncertainty, similar to the entropy, and which does not require ensembling. A quite different approach was presented by Dai et al. (2020) in the context of brain tumour segmentation: they choose the most informative example by taking a step in image space along the direction of the gradient of the loss (which is Dice), and finding the nearest neighbour of this image in a lower dimensional latent space learned with a variational autoenconder (Kingma and Welling, 2013). Finally, we would like to pinpoint that types of interaction other than clicks, contours, masks, and labels have been presented. For example, Cai et al. (2021) request user interaction at the super-pixel level, which they find to be more time efficient than polygons; they measure uncertainty with the Best-versus-Second Best (BvSB) margin Joshi et al. (2009), i.e., the ratio between the posteriors of the two most likely classes at each pixel.

A recent, comprehensive survey of deep active learning and suggestive methods for medical image segmentation can be found in Budd et al. (2021).

#### 1.2.3. Partially annotated data

An often overlooked aspect in interactive segmentation of 2D stacks is the fact that human labellers often desire to improve the segmentation of a certain structure by labelling it in additional images, but without having to label all other structures on that image. While it is trivial to train a pixel classifier using only the labelled ROIs, this approach fails to exploit the information in the unlabelled pixels, e.g., the fact that they do not belong to any of the labelled ROIs. A similar problem is faced by applications where there are multiple annotators but no gold standard due to the challenges associated with generating ground truth labels (Raykar et al., 2009; Tanno et al., 2019; Yan et al., 2010; Zhang et al., 2020a).

In the context of MAS, Commowick et al. (2012) proposed to ameliorate the effect of missing labels by adding a prior on the confusion matrices to the STAPLE algorithm (Warfield et al., 2004). Also in the context of MAS, Iglesias et al. (2015) proposed a family of probabilistic models for label fusion suited for scenarios in which different manual delineation protocols with potentially disparate structures have been used to annotate the training scans. The generative model assumes that the atlases have a hidden “fine” segmentation with all the structures present in the training data, and that the actual observed labels have been obtained by collapsing groups of hidden fine labels into more general, coarse labels. This idea has been adapted to the training of deep segmentation networks with heterogeneously labelled datasets. Essentially, the network predicts segmentations at the finest level of detail (i. e., with all possible labels), while losses (e.g., cross-entropy, Dice) are computed at the (generally coarser) level of each dataset, merging classes in the prediction as needed. In the context of cross-entropy, such generalised loss have been named “marginal cross-entropy” or “super--label-aware cross-entropy” (Kemnitz et al., 2018; Ram and Sabuncu, 2018; Fang and Yan, 2020). A generalised Dice was used by Shi et al. (2021).

### 1.3. Contribution

As previously mentioned, active learning can be used to assist the learning procedure by optimising the selection of unlabelled samples for human labelling. However, the methods described in Section 1.2.2 use querying objective functions which do not necessarily represent what the annotator is interested in, e.g., entropy rather than Dice scores. Moreover, existing techniques do not use adequate surrogates of the time it takes to label an ROI, often assigning a constant cost to every slice or structure. Therefore, there is a clear need for better proxies for the annotation effort.

In this paper, we propose a solution to these problems via a novel active learning framework for suggestive and interactive segmentation of 2D stacks of images, which exploits a number of practically useful sources of information that are often disregarded by suggestive methods. Specifically, our method:

- Requests the manual delineation of a single ROI on a single slice at every iteration – as opposed to, e.g., labelling all the structures on a slice or volume – and updates a segmentation CNN that produces dense segmentations for all slices using a principled mixed-cross entropy loss that effectively exploits partially annotated images.
- Exploits, in a principled manner, weak supervision in the form of boundary image indices for each structure, which labellers commonly use in practice (e.g., “a certain ROI first appears in image $i_{1}$ and last appears in image $i_{2}$”).
- Seeks to optimise the metric we are interested in (average Dice), rather than a proxy (e.g., entropy).
- As Kuo et al. (2018), we use a realistic surrogate for effort (tracing time) based on boundary length, but we also account for multiple ROIs and their spatial relationships, e.g., segments already labelled as part of the boundary of a neighbouring ROI.

To the best of our knowledge, this is the first method for active learning segmentation which seeks to directly optimise Dice while accounting for the boundary length of the different ROIs (including shared boundaries when available), while effectively exploiting partial annotations – and thus supporting annotations of one ROI on one 2D image at the time.

## 2. Methods

**Fig. 1.**
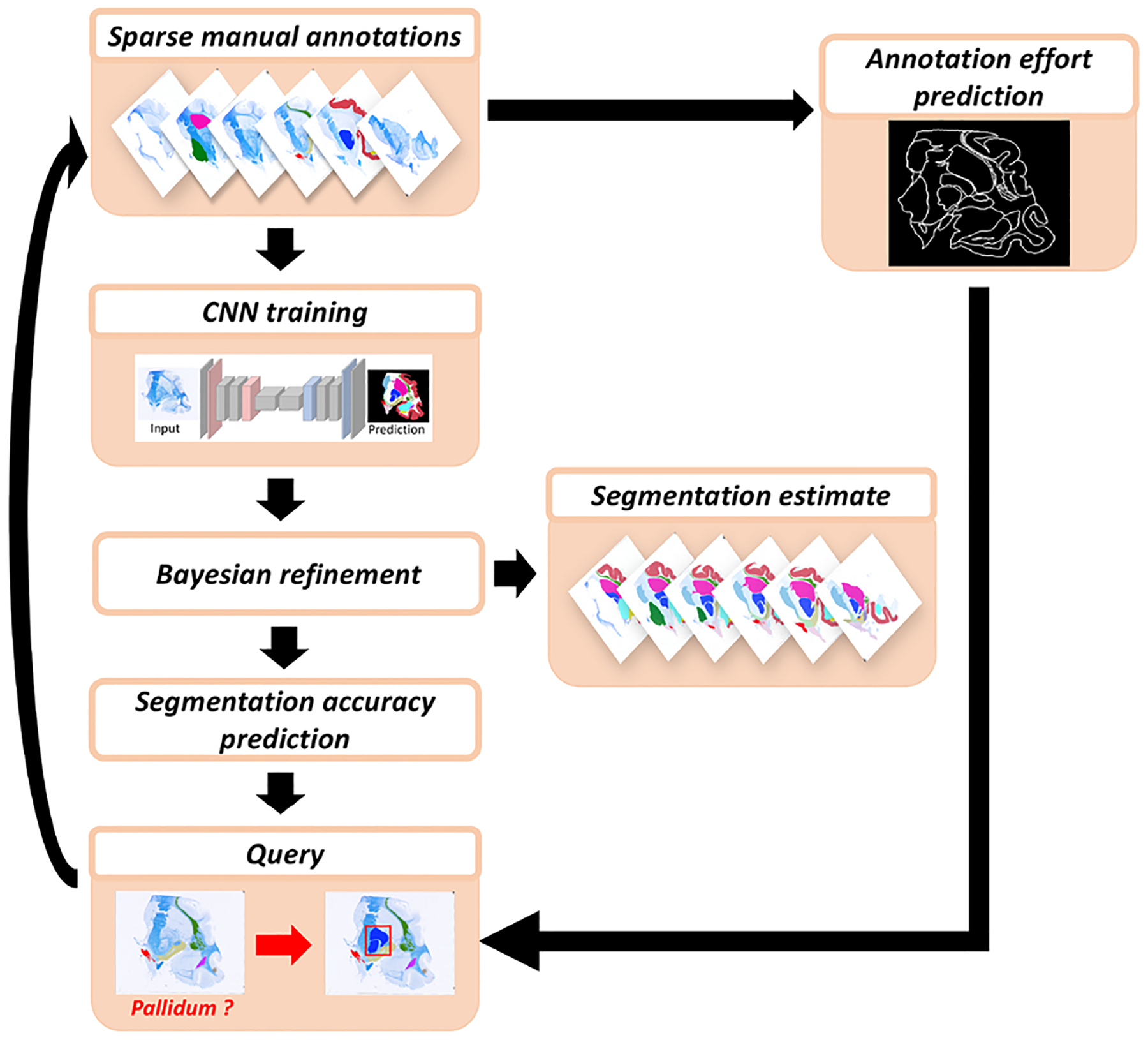
Overview of proposed method. Starting from a stack of 2D images with a minimum subset of heterogeneously manually labelled structures, we first extract statistics about the annotation effort and we train a segmentation network. We then predict the segmentation accuracy and combine it with the annotation effort prediction to formulate a query. Once a new structure is delineated, the statistics about the annotation effort is updated and the segmentation network is fine-tuned. The algorithm iterates until a target accuracy is reached.

Our proposed method aims to help the human annotator to use their time more efficiently by prompting them to delineate structures which help a segmentation CNN learn faster, i.e., using less training data. Furthermore, it requests delineations of specific ROIs on specific slices at every step, thus avoiding having to label all the ROIs on a 2D image at every iteration. The method consists of three main components (Fig. 1). First, a mixed cross-entropy segmentation loss, inspired by our previous work (Iglesias et al., 2015), which estimates probabilities from weak annotations (partially annotated images and boundary image indices). Second, a predictor for the annotation effort. And third, a novel query strategy for iterative structure suggestion that accounts for the chosen objective metric and annotation effort, including shared boundaries. The model is flexible in terms of CNN architecture and in terms of objective metric (e.g., Dice score or pixel accuracy).

### 2.1. CNN training with sparse labels and weak annotations

Let ${\left\{i_{n}(x)\right\}}_{n=1,\ldots ,N}$ be a stack of N 2D images defined on discrete coordinates x over image domains ${\left\{\Omega_{n}\right\}}_{n=1,\ldots ,N}$ (with $\Omega_{n}\subset R^{2}$), and let ${\left\{l_{n}(x)\right\}}_{n=1,\ldots ,N}$ be the corresponding segmentations (discrete label maps) that we seek to obtain, where $l_{n}(x)\in C$ (and C={1,…,C} is the set of C possible labels).

At any stage of our active learning process, the pixels in every image n in the stack can be divided into two sets: the set of manually labelled pixels $L_{n}$, and the set of unlabelled pixels $U_{n}$:

$$\begin{array}{ll} & L_{n}=\left\{x\in \Omega_{n}:x\;\text{is}\;\text{labelled}\;\text{and}\;\text{has}\;\text{label}\;l_{n}(x)\in C\right\}\\ & U_{n}=\left\{x\in \Omega_{n}:x\;\text{is}\;\text{not}\;\text{labelled}\;\text{and}\;\text{we}\;\text{know}\;\text{that}\;l_{n}(x)\in C_{n}\right\}, \end{array}$$

where $C_{n}\subseteq C$ is the set of possible labels that the unlabelled pixels in image n are compatible with (further details below). Therefore:

(1) $$\left\{\begin{array}{l} l_{n}\left(x\right)\in C,\forall x\in \Omega_{n},\\ l_{n}(x)\in C_{n},\forall x\in U_{n},\\ l_{n}(x)\notin C_{n},\forall x\in L_{n}. \end{array}\right.$$

The set $C_{n}$ is informed by two different sources. First, the set of labels present in $L_{n}$ Such labels are excluded from $C_{n}$: if an ROI has already been labelled, no pixel outside it can belong to that class anymore. The second source of information is weak supervision in the form of boundary image indices for each structure, i.e., structure c first appears in image $n=n_{c1}$ and last appears in image $n=n_{c2}$. Note that labellers routinely identify the images where each ROI is present, so exploiting this information is of high practical importance. Specifying $n_{c1}$ and $n_{c2}$ only requires two mouse clicks while the labeller is inspecting the sections and does not involve any delineation. Furthermore, if the labeller is not confident about the presence or absence of a specific structure in an image, they can add a safety margin to the boundary image indices. In sum:

$$\left\{\begin{array}{l} c\in C_{n},\;\text{if}\;\left(n\geq n_{c1}\right)\wedge \left(n\leq n_{c2}\right)\wedge \neg A_{\text{nc}},\\ c\notin C_{n},\;\text{oherwise}, \end{array}\right.$$

where $A_{nc}\in \{0,1\}$ is a binary variable that specifies whether structure c has been annotated in image n.

Now, let $f^{n}(x;\theta )={\left[f_{1}^{n},\ldots ,f_{C}^{n}\right]}^{T}$ be the probability of the pixels in image n belonging to the class c, as estimated by a CNN with parameters θ. In order to estimate θ (i.e., train the CNN), we follow our previous work in MAS (Iglesias et al., 2015) and extend the classic cross entropy function to accommodate partial annotations and weak supervision. Specifically, when the CNN is presented image $i_{n}$ during training, it will predict probabilities for all the labels c∈E. In training, we merge (sum) for every input image the predictions for the labels in the set $C_{n}$ into a generic background label. This merged “background” can be compared against the unlabelled pixels in the training set to compute the loss and backpropagate through the CNN to update its parameters θ. Specifically, the training optimises the following loss function:

(2) $$ℒ(\theta )=-\sum_{n=1}^{N}\left\{\sum_{x\in L_{n}}\text{log}f_{l(x)}^{n}(x;\theta )+\sum_{x\in U_{n}}\text{log}\left(\sum_{c\in C_{n}}f_{c}^{n}(x;\theta )\right)\right\}$$

The loss in Eq. (2) can be optimised with any standard optimiser (e. g., Adam; Kingma and Ba 2014). We note that the classical cross entropy loss is recovered if the image n is fully annotated, i.e., $U_{n}=\emptyset$, and a naive cross entropy loss ignoring missing data is obtained if only the labelled pixels are used in training, i.e., if the second term is disregarded and the known information on the unlabelled pixels is not exploited.

Once the CNN has been trained, it can be used to classify all the images in the stack. Since there is no guarantee that the prediction of the CNN will satisfy the constraints in Eq. (1), we combine the CNN output and the constraints with Bayes’s rule in order to obtain the final label probabilities at the current iteration. The probability $p_{c}^{n}(x)$ of class c at location x of image n is given by:

(3) $$p_{c}^{n}(x|\;L_{n},U_{n},{ℭ}_{n};\theta )=\text{​}\{\begin{array}{l} \delta \left(c=l_{n}\left(x\right)\right),\;\text{if}\;x\in L_{n},\\ f_{c}^{n}\left(x;\theta \right)/\underset{c^{\prime }\in {ℭ}_{n}}{\sum }f_{c^{\prime }}^{n}\left(x;\theta \right),\;\text{if}\;\left(x\in U_{n}\right)\wedge \left(c\in {ℭ}_{n}\right),\\ 0,\;\text{otherwise}. \end{array}$$

At any time of the learning process, the hard segmentation of each image n is given by:

(4) $$S^{n}\left(x\right)=\underset{c}{\text{argmax}}p_{c}^{n}\left(x|L_{n},U_{n},C_{n};\theta \right).$$

### 2.2. Annotation effort estimation

**Fig. 2.**
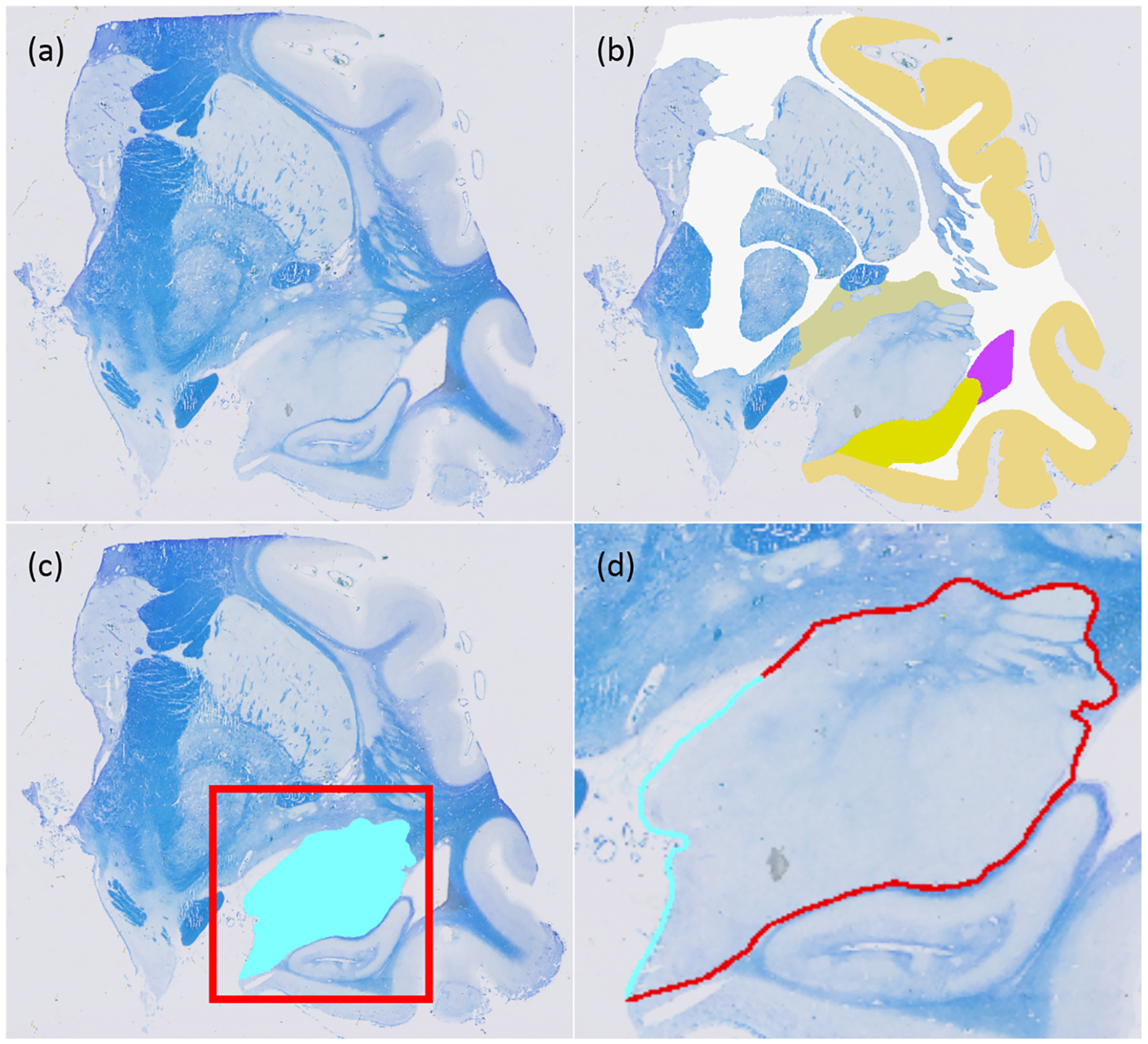
(a) Sample histological section. (b) Available manual annotations at iteration t of the active learning process. (c) Query at t+1: the algorithm has requested labelling of the amygdala on this section (in light blue). (d) The actual boundary length that needs to be labelled (in light blue) is much smaller than the perimeter of the amygdala, since most of it has already been labelled as part of neighbouring structures (in red).

To overcome the lack of knowledge about the annotation effort in terms of delineation time, we choose to use the structure boundary length as a proxy. This choice accounts for the increasing manual labelling time with the size of an ROI and the irregularity of its boundaries. For example, manually tracing a convoluted structure such as the cerebral cortex takes more time than labelling a structure with the same area but regular boundaries. Moreover, we consider neighbouring labelled ROIs when computing the boundary length, by subtracting the length of the shared boundary (see example in Fig. 2).

In order to estimate the effort (i.e., boundary length) that is required to label an ROI or a certain image, we maintain a symmetric C×C matrix $B=\left(B_{cc^{\prime }}\right)$ with the cumulative averages of the boundary lengths of the structures, as well as the averages of the shared boundary lengths between pairs of structures. Specifically, $B_{cc}$ stores the average boundary length of structure c (in pixels), and $B_{cc^{\prime }}=B_{c^{\prime }c}$ stores the average length of the boundary shared by c and $c^{\prime }$ :

$$B_{cc}=\frac{\sum_{n=1}^{N}A_{nc}\ell \left(l_{n}=c\right)}{\epsilon +\sum_{n=1}^{N}A_{nc}},$$

$$B_{cc^{\prime }}=\frac{\sum_{n=1}^{N}A_{nc}A_{nc^{\prime }}\ell \left(l_{n}=c,l_{n}=c^{\prime }\right)}{\epsilon +\sum_{n=1}^{N}A_{nc}A_{nc^{\prime }}},$$

where ℓ(M) is the boundary length (in pixels) of a binary mask M, and $\ell \left(M,M^{\prime }\right)$ is the shared boundary length of two binary masks. We note that ϵ is a small constant that places a very weak prior (around zero) on the boundary lengths. This design choice does not have any practical implications for the diagonal of B, since we assume that every ROI has been labelled once before starting the active learning (see Section 2.4 below). However, the prior keeps the estimates of the shared boundary lengths to zero for all structure pairs, until examples have been observed (i.e., c and $c^{\prime }$ labelled in the same image n). We note that adding a prior with a positive length value would encourage the active learning to explore many label pairs in the same image, which actually hinders the performance of the active learning approach described in Section 2.3 below, given the large amount of neighbouring pairs that the framework would be encouraged to explore.

Given the current estimate of B, the estimated effort (remaining boundary length $R_{nc}$) required to delineate structure c on image n is estimated as:

(5) $$R_{\text{nc}}=\left\{\begin{array}{l} B_{\text{cc}}-\sum_{c^{\prime }=1}^{C}A_{nc^{\prime }}B_{cc^{\prime }},\;\text{if}\;c\in C_{n}\\ 0,\;\text{otherwise}. \end{array}\right.$$

Note that if on a given image n all the ROIs neighbouring class c have been labelled, then the remaining required effort for that ROI c in image n is considered to be zero, i.e., $R_{nc}=0$.

### 2.3. Active learning querying strategy

Let ${\left\{n^{\left(t\right)}\right\}}_{t=1,\ldots ,T}$ and ${\left\{c^{\left(t\right)}\right\}}_{t=1,\ldots ,T}$ be, respectively, the sets of images and classes delineated by the labeller in T annotation steps, i.e., at iteration t, the the user annotated the ROI corresponding to class $c^{\left(t\right)}$ on image number $n^{\left(t\right)}$. The cumulative segmentation accuracy metric (average Dice score) D and annotation effort E are functions of these two sets:

$$D^{\left(T\right)}=g_{D}\left({\left\{n^{\left(t\right)}\right\}}_{t=1,\ldots ,T},{\left\{c^{\left(t\right)}\right\}}_{t=1,\ldots ,T}\right),$$

$$E^{\left(T\right)}=g_{E}\left({\left\{n^{\left(t\right)}\right\}}_{t=1,\ldots ,T},{\left\{c^{\left(t\right)}\right\}}_{t=1,\ldots ,T}\right).$$

Ideally, if the exact functions $g_{D}$ and $g_{E}$ were known, we could design an optimal querying strategy which, e.g., minimises the effort E required to achieve a minimum Dice score $D_{min}$, or maximises D without surpassing a maximum effort $E_{max}$ (i.e., a “labelling budget”). However, given that these functions are unknown, most active learning strategies rely on greedy approaches that seek to optimise a combined function accuracy and effort one step at the time.

Quantifying the exact annotation effort in terms of annotation time is almost always unfeasible, so surrogates for E are required. Most existing active learning methods for medical image segmentation request the labelling of an image or volume without considering the time cost that may be associated with it, and also use the number or images, patches or pixels as a proxy for time cost in evaluation (e.g., Top et al. 2011; Pace et al. 2015; Yang et al. 2017; Dai et al. 2020; Xu et al. 2021). However, ROIs require different labelling time depending on their size, shape, and spatial relation to the rest of the image. For this reason, we consider the annotation effort as being class-specific and dependent on the surrounding ROIs that may have already been labelled (as described in Section 2.2 above), such that:

$$\Delta E^{\left(T\right)}=\left(E^{\left(T\right)}-E^{\left(T-1\right)}\right)\propto R_{\text{nc}}^{\left(T\right)}.$$

A common surrogate for the function $D^{\left(T\right)}$ – or rather the increment $\Delta D^{\left(T\right)}=D^{\left(T\right)}-D^{\left(T-1\right)}$ – is the entropy of the candidate samples according to the current classifier, such that the framework queries the unlabelled examples for which the segmentation model is most uncertain. This approach is suboptimal, as the most uncertain data often comprise out-of-distribution examples. Instead, we argue that a more effective querying criterion would aim to directly optimise the expectation of a segmentation accuracy metric chosen by the labeller (Dice, in our case). To this end, we propose a simple method to predict the classifier performance at each iteration T from the performance for each ROI over previous iterations:

(6) $${\tilde{D}}_{c}^{\left(T\right)}=\alpha D_{c}^{\left(T-1\right)}+(1-\alpha )D_{c}^{\left(T-2\right)},$$

where $D_{c}$ is the Dice score for class c, and α is a smoothing parameter to balance the contribution of the current and past performance of the classifier. Note that the real $D_{c}^{\left(T-1\right)}$ is known once the labels for iteration T-1 have been provided by the annotator and the classifier has been updated, as it can simply be computed as the Dice score between the hard segmentation computed with Eq. (4) and the available manual annotations averaged across images in the stack. While this strategy lags one step behind (compared with, e.g., entropy-based approaches), it has the advantage of using the metric of interest (Dice) directly, rather than a surrogate.

Given our estimates of Dice and effort, we propose to maximise the ratio between their increments at every iteration:

$$\left\{{\hat{n}}^{\left(T\right)},{\hat{c}}^{\left(T\right)}\right\}=\underset{n,c}{\text{argmax}}\frac{\Delta D_{c}^{\left(T\right)}}{\Delta E^{\left(T\right)}}=\underset{n,c}{\text{argmax}}\frac{\Delta D_{c}^{\left(T\right)}}{R_{\text{nc}}^{\left(T\right)}}.$$

In practice, we have found the estimates of Dice improvement $\Delta D_{c}^{\left(T\right)}$ to be too noisy. Better results are obtained by replacing it by the complement of the Dice score, i.e., the room for improvement, such that our final active learning criterion is:

(7) $$\left\{{\hat{n}}^{\left(T\right)},{\hat{c}}^{\left(T\right)}\right\}=\underset{n,c}{\text{argmax}}\frac{\left(1-{\tilde{D}}_{c}^{\left(T\right)}\right)}{R_{nc}^{\left(T\right)}}.$$

### 2.4. Implementation details

#### 2.4.1. Network architecture

Our implementation relies on a simple fully convolutional network (FCN) built on top of a VGG-16 architecture (Simonyan and Zisserman, 2014). Skip connections were added between lower and higher layers, enabling dense prediction at input resolution; further details can be found in Long et al. (2015).

#### 2.4.2. Network training

Training sought to minimise Eq. (2), combined with a L2-norm penalty on the network weights (with relative weight 0.0001). During training, images and labels were augmented with random geometric (rotation, translation, scaling, shearing, non-linear deformation) and intensity (brightness, contrast) perturbations, randomly cropped to patches of size [128, 128], and min-max normalised. Nonlinear deformation was achieved by independently sampling a bivariate Gaussian distribution (diagonal covariance, σ=4 pixels) with (x,y) shifts at a set of control points, located on regular grid with 5 pixel spacing; a dense field is obtained by interpolating the shift between the control points. The rest of augmentation parameters were sampled from uniform distributions, with minimum and maximum values summarised in Table 1.


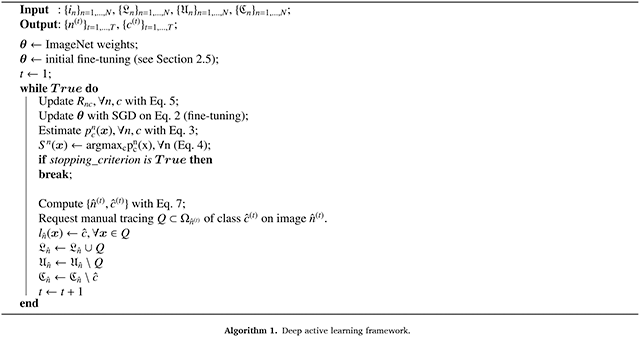


The network weights were optimised with a stochastic gradient descent algorithm with momentum 0.9 (Qian, 1999) and batch size 8. We follow the experimental setup of Long et al. 2015 and set the learning rate to 0.05 for the initial fine-tuning and to 0.005 for the further fine-tuning, as they provided good performance in previous work on histology (Atzeni et al., 2018). We trained the initial network for 3,000 epochs (approximately 10,000–20,000 iterations, depending on the size of the stack), after which we observed minimal change in the loss and no perceptible difference in the outputs. During the active learning, we fine-tuned the network for 10 further epochs at each iteration t. All models were trained on a NVIDIA Tesla V100 GPU.

### 2.5. Summary of algorithm

The proposed method is summarised in Algorithm 1. It requires an initial set of manual annotations ${\left\{L_{n}\right\}}_{n=1,\ldots ,N}$, which contains at least one segmentation of each structure somewhere in the stack of images, and the set of compatible labels for each image ${\left\{C_{n}\right\}}_{n=1,\ldots ,N}$, acquired through weak annotation. The algorithm starts by initialising the VGG-16 portion of the network with publicly available weights resulting from training on ImageNet (Deng et al., 2009). We then make an initial fine-tuning of the network with Eq. (2) (i.e., in a semi-supervised fashion) using all the available partial information: the manual annotations in the labelled images, and the boundary image indices for all ROIs. This semi-supervised strategy, combined with the ImageNet initialisation, makes training robust against overfitting, despite the small amount of labelled pixels.

After initialising the network, the framework updates the estimated effort according to Eq. (5), and produces a segmentation prediction (Eq. (4)) which is the result of the combination of the CNN prediction and the constraints derived by the information contained in the partial labelling and weak annotation, as described in Eq. (3). The segmentation performance of the previous iterations and the estimated effort are then used to formulate a query (i.e., request the labelling of an ROI on a given image), according to the selection criteria described in Eq. (7). The aforementioned steps are repeated until a stopping criterion is reached. Possible stopping criteria include reaching a desired average Dice score or a maximum amount of manual annotation effort. We note that the only hyperparameter of our active learning model is the smoothing term α (Eq. (6)), which we set to 0.5 in all experiments below.

## 3. Experiments and results

This section presents two sets of experiments. The first set aims to compare our framework with three baseline methods that are commonly used in interactive segmentation with active learning: random, slice-wise uniform, and entropy based selection. The second set of experiments consists of an ablation study that explores how different elements of the method contribute to the final performance of our active learning framework. Both sets of experiments are performed on two different datasets: a synthetic dataset consisting of artificially deformed brain MRI slices, and a real dataset of thoroughly labelled human brain histology.

### 3.1. Datasets

#### 3.1.1. Synthetic MRI dataset

The synthetic dataset was generated by artificially deforming the publicly available 3D MRI dataset from the MICCAI 2013 Challenge “Workshop on Segmentation: Algorithms, Theory and Applications (SATA)” (Asman et al., 2013). The dataset consists of 35 T1-weighted brain scans acquired on a 3T scanner with an MP-RAGE sequence at 1mm isotropic resolution. Fourteen structures were labelled by experts in coronal plane: left and right amygdala, caudate, accumbens, hippocampus, putamen, thalamus and pallidum. We augmented these labels with left and right white matter and cerebral cortex segmentations obtained with FreeSurfer (Fischl et al., 2002; Fischl, 2012). Labels for contralateral structures were merged.

From these 3D MRI volumes and corresponding segmentations, we synthesised a stack of coronal images as follows: *(i)* Removing the coronal slices of the volume not containing diencephalon structures; *(ii)* Sparsifying the volume, dropping four every five coronal slices (i.e., 5 mm spacing); and *(iii)* Deforming the remaining coronal slices (and corresponding segmentations) with 2D deformation fields generated independently for each slice. The 2D fields were generated as the composition of a similarity and a nonlinear transform. The similarity component is a combination of random rotations, translations and log-scalings, all sampled from zero-mean Gaussian distributions, with standard deviations of 10∘, 10 pixels, and 0.1, respectively. The nonlinear component is sampled with the same method we used for nonlinear augmentation in training (Section 2.4.2). The final stacks had an average of 24 images, with 256 × 256 pixels each.

#### 3.1.2. Histology dataset

We also present results for 15 stacks of 2D histology sections of human brain tissue. The tissue used for this experiment was donated for research to the Queen Square Brain Bank for Neurological Disorders. The dataset consists of 15 coronal blocks dissected from three hemispheres from three different human brains. Each block was processed for paraffin wax embedding, and subsequently sectioned with a sledge microtome at 25μm thickness (see Mancini et al. 2020 for further details on the tissue processing). The sections were mounted on 74 × 52mm glass slides, stained with Luxol fast blue with cresyl violet, and digitised at 3.97μm resolution. For the purposes of this article, we downsampled the images to 160μm resolution (approximately 400 × 300 pixels), as a compromise between detail and computational requirements. Trained research assistants, supervised by expert neuroanatomists, manually segmented one section every ten in subcortical blocks (~40 labelled sections per block), and one section every twenty in cortical blocks (~20 per block). Blocks have 9 fully labelled sections and 70 different ROIs on average (266 unique ROIs in the dataset in total); detailed descriptions of the blocks and ROIs can be found in the Supplementary Material.

### 3.2. Experimental setup

#### 3.2.1. Simulation of user interactions

User interaction was simulated as follows. The initial set of manual annotations $\left({\left\{L_{n}\right\}}_{n=1,\ldots ,N},{\left\{U_{n}\right\}}_{n=1,\ldots ,N},{\left\{C_{n}\right\}}_{n=1,\ldots ,N}\right.)$ was created by picking for labelling a central section for each ROI. We simulate the user’s choice of central section by taking the actual central image for each ROI and randomly picking one within 3 sections of it. From that point on, the results of the queries are simulated by simply taking the ground truth segmentation for the queried section / ROI pair. Since every image stack in the real histology dataset has different visible ROIs, we trained a separate, dedicated model for every stack with Algorithm 1. The cost of labelling is simply the total boundary length of the delineated ROIs, accounting for shared boundaries.

#### 3.2.2. Competing methods

We compare the proposed method against three commonly used baselines:

**Random selection** (henceforth “**rand**”): this model follows the same steps described in Section 2.5, except for the active learning stage where both image and structure indices are selected randomly instead of with Eq. (7). Each image and structure in the unlabelled pool has an equal probability to be queried.

**Slice-wise uniform** (henceforth “**uniform**”): in this model, the manual annotator delineates all ROIs (in random order) in one section, before proceeding to the next. Sections are labelled in an order that minimises, at every step, the maximum number of consecutive unlabelled sections (ties are broken randomly).

**Entropy** (henceforth “**H**”): the framework suggests the structure c within the image n for which the entropy is maximal, i.e.,

(8) $$\{\hat{n},\hat{c}\}\;=\underset{n,c}{\text{argmax}}H_{nc},\text{where}\;H_{nc}\;=-\frac{1}{\left|\Omega \right|}\sum_{x\in \Omega }p_{c}^{n}\left(x|L_{n},U_{n},C_{n};\theta \right)logp_{c}^{n}\left(x|L_{n},U_{n},C_{n};\theta \right).$$

**Entropy with Monte Carlo dropout** (henceforth **‘H_dropout**”): the entropy given by Eq. (8) is often underestimated as it does not account for the uncertainty in the model (i.e., in its weights). In order to obtain more realistic estimates of the entropy, we recompute the entropy with label probabilities obtained with Monte Carlo dropout (Gal and Ghahramani, 2016), i.e., replacing $p_{c}^{n}$ with:

(9) $$p_{c}^{n}\leftarrow \frac{1}{S}\sum_{s=1}^{s}p_{c}^{n}\left(x|L_{n},U_{n},C_{n};\theta_{s}\right),$$

where S=20 is the number of Monte Carlo samples, each yielding network weights $\theta_{s}$ obtained with dropout at testing with probability 0.5.

#### 3.2.3. Ablation study

In order to quantitatively assess the contribution of each element in the proposed active learning framework, we perform an ablation study where, starting from the proposed method, we remove or add model components.

The compared models are the following:

##### BDα (boundary length, Dice, and α).

This is our proposed approach, where CNN training is performed using Eq. (2), and the query strategy follows Section 2.3.

##### Dα (Dice, and α).

We further ablate the boundary length, i.e., we set $B_{cc^{\prime }}=0,\forall c\neq c^{\prime }$.

##### $BD\alpha_{-}CE$ (BD α with cross entropy).

We investigate the effect of removing the proposed loss (Eq. (2)) and replacing it with a standard cross entropy loss.

##### HBDα(BDα with entropy term).

CNN training is performed using Eq. (2). We add to the querying criterion a term with the entropy of the ROI and section at hand according to the current classifier, such that the framework optimises:

$$\left\{{\hat{n}}^{\left(T\right)},{\hat{c}}^{\left(T\right)}\right\}=\underset{n,c}{\text{argmax}}\frac{\left(1-{\tilde{D}}_{c}^{\left(T\right)}\right)H_{\text{nc}}^{\left(T\right)}}{R_{\text{nc}}^{\left(T\right)}},$$

where $H_{nc}^{\left(T\right)}$ is calculated with Eq. (8).

##### **HD α** (Dice and α with entropy term).

CNN training is performed using Eq. (2). We now reinstate α and ablate the annotation effort estimate $R_{nc}^{\left(T\right)}$, such that the framework optimises:

$$\left\{{\hat{n}}^{\left(T\right)},{\hat{c}}^{\left(T\right)}\right\}=\underset{n,c}{\text{argmax}}\left(1-{\tilde{D}}_{c}^{\left(T\right)}\right)H_{\text{nc}}^{\left(T\right)}.$$

##### **HB** (entropy and boundary length).

CNN training is performed using Eq. (2). For the query strategy we ablate the accuracy prediction and replace it with the entropy, i.e., we optimise:

$$\left\{{\hat{n}}^{\left(T\right)},{\hat{c}}^{\left(T\right)}\right\}=\underset{n,c}{\text{argmax}}\frac{H_{\text{nc}}^{\left(T\right)}}{R_{\text{nc}}^{\left(T\right)}}.$$

### 3.3. Results

**Fig. 3.**
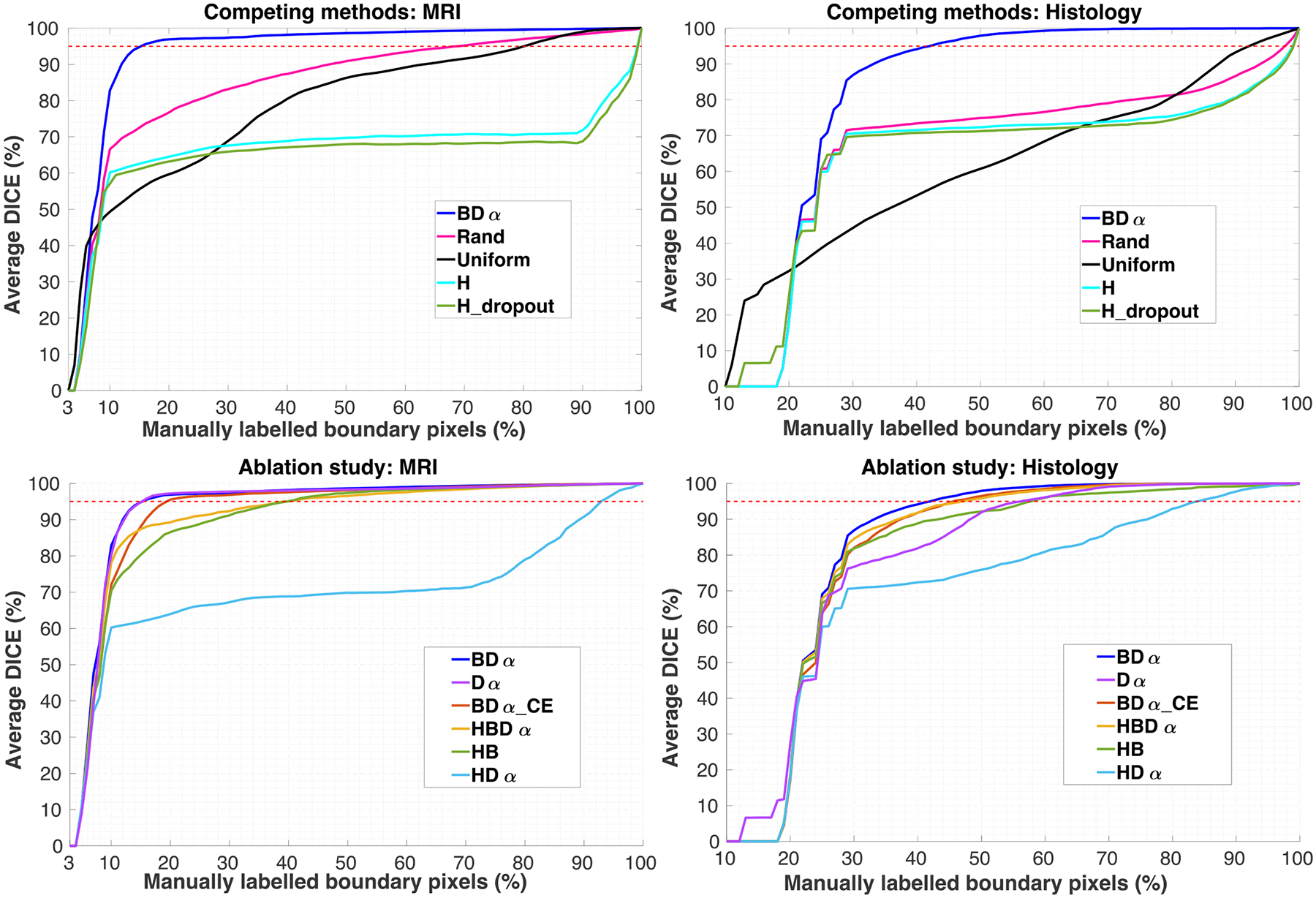
Average Dice (computed across stacks and ROIs) against the percentage of boundary pixels manually labelled. The top row shows the results for the competing methods, whereas the bottom row refers to the ablation study. The dotted red line indicates 95% Dice. Note that the initial labelling effort needed to bootstrap the algorithm (i.e., labelling each ROI once) is approximately 3% of the boundary length in the MRI dataset and 20% in the histology (higher, due to the larger number of ROIs). The results for “Rand” are averages over five runs.

The results of the first set of experiments, which compares the performance of the proposed method with three commonly used baselines, are shown in the top row of Fig. 3. The graphs show the average Dice score (computed across stacks and ROIs) against the percentage of boundary pixels manually labelled for the synthetic (MRI) and real dataset (histology), respectively. The plots for the random strategy are averages over five runs. The results are consistent across the two datasets: the proposed method generally climbs faster than the competing baselines and outperforms them all across the range of labelling efforts.

The method based purely on entropy (which is widely used in the active learning literature) is heavily penalised by its bias towards bigger ROIs (please see Figures S4 and S5 in the supplement): labelling such structures requires a larger labelling effort, and neglecting small ROIs has a detrimental effect on the average Dice. The corresponding curves plateau at 65–70% Dice for the two datasets, and only increase further when one starts labelling the smaller ROIs towards the end. Furthermore, the addition of Monte Carlo dropout does not affect this trend, as it suffers from the same bias towards larger ROIs; the results with and without Monte Carlo sampling are very similar for both datasets.

The method based on random selection does not have these problems, as it is not biased towards any structure or section. However, that also means that it is unable to exploit knowledge on structure size, shared boundaries, etc. Neglecting such information seems to be particularly inefficient in the histology dataset, due to the higher number of ROIs - and thus more complex distribution of neighbouring ROIs and boundary lengths.

In terms of ROIs, the slice-wise uniform approach only has a slight bias towards structures that appear on more sections. However, it has (by construction) a very high spatial bias, labelling all ROIs in a section before proceeding to the next. While this is an effective way of discovering neighbouring relationships, it also precludes sampling of different ROIs in different parts of the stack, and also leads to oversampling of large (and thus expensive) ROIs. As a result, its performance is worse than that of the random selection method on both datasets.

In contrast, the proposed method mitigates the effect of structure size by considering the typical boundary length. Moreover, it further utilises knowledge on shared boundaries and expected Dice score improvements, which the competing methods do not exploit. This yields great increases in the Dice scores that can be achieved with a certain labelling budget (Tables 2 and 3), or, alternatively, great reductions in the labelling effort that is required to achieve a target Dice score (Tables 4 and 5). For example, our approach reduces by approximately 75% the effort that is required to achieve a 90% or 95% Dice compared with the second-best method (random selection) in the MRI dataset. The reduction is approximately 60% in the histology dataset.

The bottom row of Fig. 3 compares our approach with the ablated variants. Each model element (mixed cross entropy, boundary length module, estimation of Dice improvement) contributes to reduce the effort needed to reach a predefined Dice score, in a statistically significant fashion (Tables 2–5). Of these elements, the boundary length estimation is crucial for the performance of the proposed algorithm on both datasets – but particularly for the histology, where there is a complex network of neighbourhood relationships. It is also apparent from the graphs that the entropy term is detrimental to the performance, even when used in combination with other elements.

**Fig. 4.**
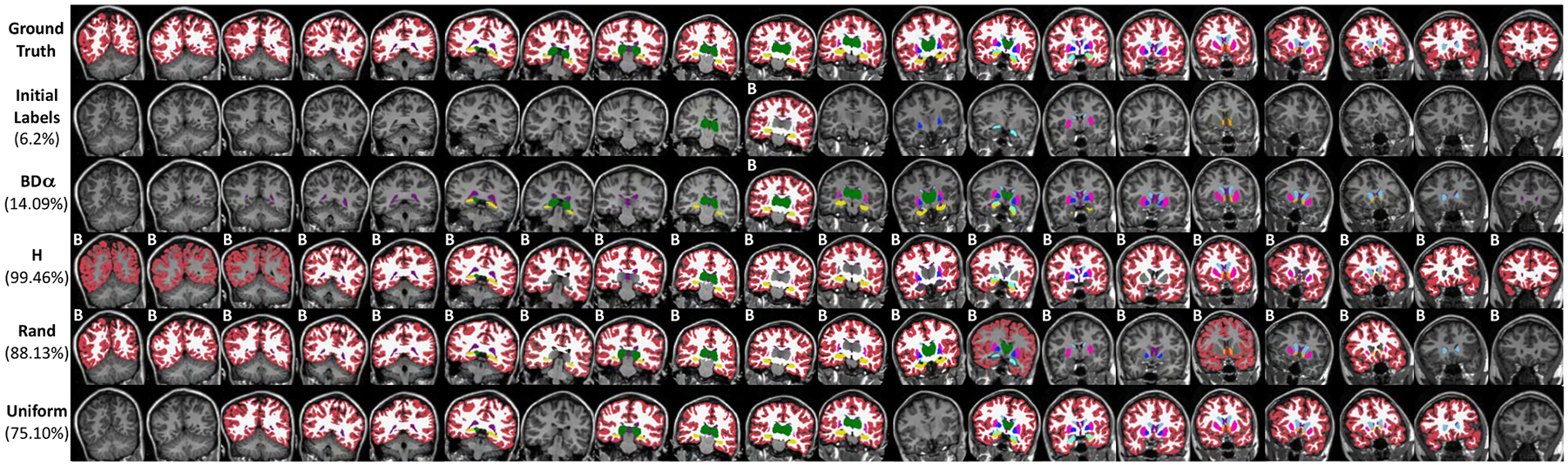
Qualitative results for a synthetically deformed MRI stack comparing the proposed framework with three commonly used baselines: entropy based (H), random, and uniform. The figure represents a snapshot of the manually annotated ROIs when the different methods reach a Dice score of 95%, starting from the same set of manual delineations. From top to bottom the rows show the ground truth labels which have been synthetically created, the initial training set consisting of manual annotation of one instance for each structure in the image stack, the proposed framework and the competing baselines. In parenthesis we report the corresponding manual effort (in % of the total effort). The label B indicates the background has been annotated; we omit this for the ground truth and the slice-wise uniform case as they require whole slice annotation.

**Fig. 5.**
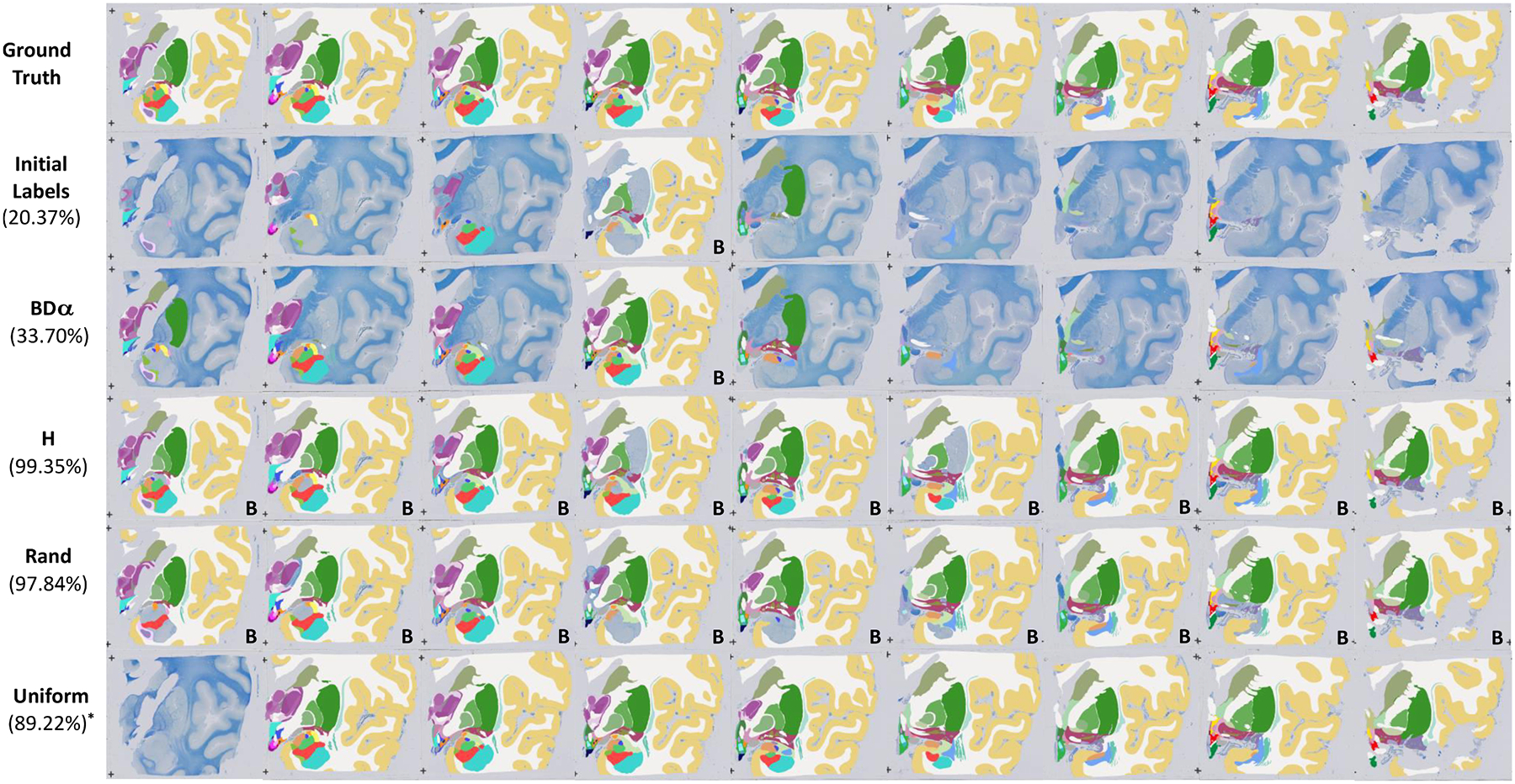
Qualitative results for a histology block comparing the proposed framework with three commonly used baselines: entropy based (H), random, and slice-wise uniform. The figure represents a snapshot of the manually annotated ROIs when the different methods reach a Dice score of 95% starting from the same set of manual delineations. From top to bottom the rows show the ground truth labels, the initial training set consisting of manual annotation of one instance for each structure in the block, the proposed framework and the competing baselines. In parenthesis we report the corresponding manual effort (in % of the total effort). In the uniform case labelling all sections but one (corresponding to an effort of 89.22%) achieves 92.20% Dice, i.e., still below the 95% standard used for the other methods. The label B indicates the background has been annotated; we omit this for the ground truth and the slice-wise uniform case as they require whole slice annotation.

Finally, Figs. 4 and 5 qualitatively compare the results of the queries for the different strategies, on the MRI and histology datasets, respectively. Each figure shows a snapshot of the manually annotated ROIs when a Dice score of 95% has been reached from the same initial set of delineations, which gives an idea of: what ROIs were prioritised and on what slices; and how much delineation effort was required to achieve 95% Dice (similar figures for 90% Dice score are shown in Figures S1 and S2 in the Supplementary Material). One can observe how the proposed method does not require the annotation of large structures like the cerebral cortex or white matter (besides the initial labels), considerably reducing the annotation effort. Furthermore, we can observe how our method finds a balance between spreading ROI annotations across the annotated; we omit this for the ground truth and the slice-wise uniform case as they require whole slice annotation. stack (to obtain information on many images) and querying clusters of ROIs (to take advantage of the contour of neighbouring structures that have already been labelled). This is in contrast with the baselines, which waste a lot of labelling effort on larger structures that do not contribute much to the average Dice.

## 4. Discussion and conclusion

In this study, we have presented a novel active learning framework for heterogeneously labelled stacks of biomedical images, which leverages on a realistic surrogates of the annotation effort and of the accuracy measure one aims to optimise (Dice). Our framework exploits partial annotations, weak supervision (in the form of boundary indices), and realistic estimates of class- and section-specific annotation effort in order to greatly reduce the time it takes to produce accurate segmentations (e.g., Dice > 0.90 or Dice > 0.95) for large histological datasets.

Our results on synthetic data show that our framework significantly outperforms three commonly used baselines (entropy, random, and uniform), decreasing the labelling effort that is required to achieve accurate segmentations by 70%. Although the real histology dataset is intrinsically more complex due to the number of structures to be labelled and the characteristics of the images themselves, the results follow the same trend in these experiments (60% reduction) and confirm the ability of our method to produce accurate segmentations with lower annotation effort on real data with real-world artifacts (e.g., folding and tearing of tissue).

Considerably reducing labelling times is crucial when annotating histology at large scale. For example, we are currently using semi-automated techniques to build a high resolution atlas of the human brain, based on approximately 5000 sections that need to be labelled. Our current approach is based on uniform labelling (labelling one section every four in each block), which approximately accounts for 5000–10,000 h of manual labour. The results in this paper suggest that a reduction of up to 60% in labelling time may be achieved with our proposed technique, which would represent an approximate saving of 90 weeks of work.

The experiments in this article have used boundary length as a proxy for labelling cost in the evaluation. Ideally, one would instead use actual labelling time measured in ideal circumstances. However, attaining such ideal circumstances is very difficult in practice. Fatigue in the annotator and memory bias when labelling the same dataset twice with different methods both introduce noise in the measurements. Moreover, timing experiments are not reproducible. Boundary length is immune to these issues, and is also a better proxy for labelling time than the commonly used number of images, volumes or patches, as explained in Section 2.3.

Future work will focus on exploring statistical priors on the different variables in the model. For example, one could build a model of the boundary length B based on knowledge derived from previous cases or from anatomy (e.g., “thalamus and amygdala are never neighbours”). We will also investigate the possibility of improving the efficiency of the algorithm by allowing the user to *correct* the CNN segmentation for an ROI on a given image, which may be more efficient than requesting manual delineation from scratch – particularly if the corrections are provided with an interactive algorithm. Further improvements in efficiency may be achieved by reusing models actively trained on one dataset when segmenting a similar dataset, e.g., of the same modality and with the same visible structures.

As high-resolution histological datasets become increasingly available in atlasing and brain mapping, we believe that approaches like the one presented in this paper will be crucial for generating ground truth labels at scale.

## Supplementary Material

list of brain areas

supplementary figures

## Figures and Tables

**Table 1:** Minimum and maximum values of the uniform distributions for each of the CNN augmentation parameters. $\left[a_{\text{rot}},b_{\text{rot}}\right]$ correspond to rotation angles (in degrees), $\left[a_{\text{trans}}\right.,\left.b_{\text{trans}}\right]$ correspond to translations (in pixels), $\left[a_{sc},b_{sc}\right]$ correspond to scalings (in logarithmic domain), $\left[a_{sh},b_{sh}\right]$ correspond to shearing angles (in degrees), $\left[a_{br},b_{br}\right]$ correspond to image brightness (in [0,255] scale), and $\left[a_{br},b_{br}\right]$ correspond to image contrast.

$a_{\text{rot}}$	$b_{\text{rot}}$	$a_{\text{trans}}$	$b_{\text{trans}}$	$a_{sc}$	$b_{sc}$	$a_{sh}$	$b_{sh}$	$a_{br}$	$b_{br}$	$a_{\text{contr}}$	$b_{\text{contr}}$
−10	10	−10	10	0.8	1.2	−10	10	−20	20	0.8	1.25

**Table 2:** Synthetic MRI data. Achieved Dice (in %) at five different labelling budgets (in % of total boundary length).

Bound. length	15%	20%	40%	70%	90%
BDα	***94.78* ± *2.41***	***96.88* ± *0.69***	***98.16* ± *0.37***	***99.32* ± *0.11***	***99.79* ± *0.03***
BDα_CE	88.07 ± 6.35	95.53 ± 1.26	97.56 ± 0.39	99.10 ± 0.20	99.79 ± 0.03^†^
Dα	94.81 ± 2.53	97.19 ± 0.38	98.02 ± 0.28	98.93 ± 0.16	99.72 ± 0.05
HBDα	87.34 ± 3.89	89.23 ± 3.53	95.05 ± 1.69	98.41 ± 0.44	99.65 ± 0.11
HB	80.30 ± 9.90	86.53 ± 8.47	95.02 ± 3.88	98.78 ± 1.47	99.58 ± 0.57
HDα	61.83 ± 5.66	63.95 ± 5.57	68.81 ± 5.68	71.30 ± 5.24	90.82 ± 3.14
H	62.57 ± 5.81	64.51 ± 5.87	68.84 ± 6.19	70.70 ± 5.33	71.83 ± 5.62
H_dropout	61.11 ± 4.9	63.19 ± 5.14	67.09 ± 5.13	68.14 ± 4.84	68.73 ± 5.67
Rand	75.52 ± 7.38	76.70 ± 6.80	87.35 ± 4.22	95.19 ± 2.23	98.33 ± 1.15
Uniform	55.55 ± 6.53	59.68 ± 6.47	80.45 ± 4.61	91.56 ± 2.79	98.99 ± 0.86

The dagger † indicates that p>0.001 for a Wilcoxon signed-rank test comparing the method at hand against ours.

**Table 3:** Histology data. Dice (in %) achieved at five different labelling budgets (in % of total boundary length).

Bound. length	35%	40%	50%	70%	90%
BDα	***91.67* ± *6.57***	***94.44* ± *5.29***	***98.04* ± *2.22***	***99.79* ± *0.22***	***99.95* ± *0.04***
BDα_CE	87.99 ± 8.59	92.17 ± 6.31	96.69 ± 3.68	99.64 ± 0.39^†^	99.96 ± 0.02^†^
Dα	79.32 ± 13.00	81.90 ± 12.96	91.94 ± 10.12	99.14 ± 2.59	99.96 ± 0.03
HBDα	89.04 ± 8.26^†^	92.21 ± 7.17	96.04 ± 3.96	99.38 ± 0.92^†^	99.92 ± 0.10^†^
HB	85.91 ± 8.23	89.49 ± 7.63	92.68 ± 5.50	97.59 ± 2.13	99.23 ± 0.73
HDα	71.28 ± 9.44	72.29 ± 9.18	75.57 ± 8.11	86.77 ± 6.47	97.77 ± 2.14
H	71.10 ± 9.48	71.55 ± 9.28	72.34 ± 9.09	73.92 ± 8.68	80.80 ± 6.85
H_dropout	70.25 ± 9.95	70.82 ± 9.65	71.27 ± 9.42	72.91 ± 9.05	80.36 ± 7.15
Rand	72.76 ± 9.02	73.70 ± 8.74	75.25 ± 8.38	79.40 ± 7.30	86.85 ± 5.46
Uniform	51.16 ± 13.64	55.24 ± 13.15	62.46 ± 13.84	75.80 ± 14.64	93.53 ± 5.49

The dagger † indicates that p>0.001 for a Wilcoxon signed-rank test test comparing the method at hand against ours.

**Table 4:** Synthetic MRI data. Manual labelling effort (in % of total boundary length) needed to reach 90% and 95% Dice score. All p-values estimated with the Wilcoxon signed-rank test comparing the method at hand against ours are below 0.001.

Dice	90%	95%
BDα	***11.63* ± *2.40***	***14.97* ± *2.29***
BDα_CE	15.71 ± 2.20	18.86 ± 2.70
Dα	12.00 ± 1.88	14.86 ± 1.73
HBDα	21.49 ± 7.13	39.31 ± 8.44
HB	26.77 ± 12.07	35.06 ± 13.68
HDα	88.97 ± 2.29	93.11 ± 1.20
H	98.71 ± 0.89	99.60 ± 0.50
H_dropout	99.20 ± 0.68	99.74 ± 0.44
Rand	45.89 ± 11.44	66.01 ± 11.25
Uniform	62.17 ± 9.42	78.77 ± 5.54

**Table 5:** Histology data. Manual labelling effort (in % of total boundary length) needed to reach 90% and 95% Dice score.

Dice	90%	95%
BDα	***33.27* ± *8.39***	***38.80* ± *10.50***
BDα_CE	37.06 ± 9.90	42.60 ± 12.19
Dα	44.33 ± 14.68	48.20 ± 16.09
HBDα	36.67 ± 10.49^†^	43.27 ± 13.84^†^
HB	42.93 ± 13.48	52.67 ± 17.87
HDα	70.67 ± 15.74	79.87 ± 17.17
H	92.07 ± 20.01	93.93 ± 20.47
H_dropout	92.00 ± 21.91	93.47 ± 22.27
Rand	87.47 ± 19.35	92.57 ± 19.58
Uniform	85.07 ± 7.64	91.00 ± 4.31

The dagger † indicates that p>0.001 for a Wilcoxon signed-rank test comparing the method at hand against ours.

## Data Availability

The data that has been used is confidential.
