# Supplementary material for: Deep active learning for suggestive segmentation of biomedical image stacks via optimisation of Dice scores and traced boundary length
Source: Med Image Anal. Author manuscript; Available in PMC 2024 Nov 29. (PMC11605667; doi:10.1016/j.media.2022.102549)
Supplement: list of brain areas [file NIHMS2036326-supplement-list_of_brain_areas.docx]

Below we list all the blocks used in our experiments, and the regions annotated therein.

The blocks follow the nomenclature [case]_[slice].[block] (e.g., P41-16_A1.2 refers to the hemisphere P41-16, and block 2 within slice A1). An example of block cut planning and corresponding nomenclature can be found in Figure S3 of the Supplementary Material.

We then indicate, per each block, the label index and the label name of the annotated regions as they appear in a customised look up table based on the Allen human brain atlas^[1]^ .

^[1]^ Allen Brain Atlas website: <https://human.brain-map.org/> .

- P41-16_A1.2
  - 0 background
  - 1 cerebral_cortex
  - 2 white_matter_of_forebrain
  - 3 peri-caudate_ependymal_and_subependymal_zone
  - 4 putamen
  - 5 anterior_olfactory_nucleus
  - 6 claustrum
  - 7 medial_septal_nucleus
  - 8 basal_nucleus_of_meynert
  - 9 diagonal_band
  - 10 bed_nucleus_of_stria_terminalis
  - 11 body_of_caudate
  - 12 external_segment_of_globus_pallidus
  - 13 ventral_pallidus
  - 14 lateral_olfactory_area
  - 15 nucleus_of_diagonal_band
  - 16 substantia_innominata
  - 17 darkly-stained_nanocellular_islands_of_si
  - 18 anterior_commissure
  - 19 lightly-stained_parvocellular_islands_of_si
  - 20 preoptic_region_of_hypothalamus
  - 21 lateral_preoptic_area
  - 22 median_preoptic_nucleus
  - 23 piriform_cortex
  - 24 nucleus_subputaminalis
  - 25 suprachiasmatic_nucleus
  - 26 optic_chiasm
  - 27 organum_vasculosum_laminae_terminalis
  - 28 temporal_claustrum
  - 29 supraoptic_region_of_hypothalamus
  - 30 stria_terminalis
  - 31 medial_preoptic_nucleus
  - 32 anterior_hypothalamic_nucleus
  - 33 paraventricular_nucleus_of_hypothalamus
  - 34 supraoptic_nucleus
  - 35 fornix
  - 36 stria_medullaris_of_thalamus
  - 37 internal_segment_of_globus_pallidus
  - 38 periventricular_nucleus__supraoptic_portion
  - 39 optic_tract
  - 40 supraoptic_dicussation
  - 41 amygdaloid_complex
  - 42 anterior_amygdaloid_area
  - 43 basolateral_nucleus_(basal_nucleus)
  - 44 ventral_division_of_basomedial_nucleus
  - 45 thalamus
  - 46 anterodorsal_nucleus_of_thalamus
  - 47 anteroventral_nucleus_of_thalamus
  - 48 parataenial_nucleus_of_thalamus
  - 49 parvocellular_division_of_va
  - 50 fasciculosus_nucleus_of_thalamus
  - 51 paraventricular_nucleus
  - 52 zona_incerta
  - 53 periventricular_nucleus__tuberal_portion
  - 54 juxtaparaventricular_lateral_hypothalamic_area
  - 55 dorsomedial_hypothalamic_nucleus
  - 56 lateral_hypothalamic_area__tuberal_part
  - 57 accessory_secretory_cells_of_lateral_hypothalamus
  - 58 pallidohypothalamic_area
  - 59 ventral_subdivision_of_coa
  - 60 paralaminar_nucleus
  - 61 amygdalocortical_(corticoamygdaloid)_transition_area
  - 62 dorsal_part_of_ventromedial_hypothalamic_nucleus
  - 63 ventral_part_of_ventromedial_hypothalamic_nucleus
  - 64 arcuate_nucleus_of_hypothalamus
  - 65 inferior_thalamic_peduncle
  - 66 anteromedial_nucleus_of_thalamus
  - 67 reuniens_nucleus_(medioventral_nucleus)_of_thalamus
  - 68 reticular_nucleus_of_thalamus
  - 69 central_part_of_ventromedial_hypothalamic_nucleus
  - 70 tuberomammillary_nucleus
  - 71 lateral_tuberal_nuclei
  - 72 ventral_medial_nucleus_of_thalamus
  - 73 posterior_hypothalamic_nucleus
  - 74 mammillothalamic_tract
  - 75 medial_subdivision_of_central_nucleus
  - 76 lateral_subdivision_of_central_nucleus
  - 77 rostral_subdivision_of_medial_nucleus
  - 78 magnocellular_(medial)_division_of_md
  - 79 magnocellular_division_of_va
  - 80 central_medial_nucleus_of_thalamus
  - 81 paracentral_nucleus_of_thalamus
  - 82 intermediodorsal__nucleus_of_thalamus
  - 83 dorsal_hypothalamic_area
  - 84 bundle_x
  - 85 lateral_hypothalamic_area_posterior_part
  - 86 mammillary_peduncle
  - 87 amygdalohippocampal_area
  - 88 central_dorsal_nucleus_of_thalamus
  - 89 supramammillary_nucleus
  - 90 lateral_part_of_medial_mammillary_nucleus
  - 91 medial_part_of_medial_mammillary_nucleus
  - 92 basal_division_of_medial_mammillary_nucleus
  - 93 lateral_mammillary_nucleus
  - 94 central_part_of_medial_division_of_md
  - 95 parvocellular_(central)_division_of_md
  - 96 rostral_division_of_vl
  - 97 rostral_subiculum
  - 98 dorsal_subdivision_of_vlc
  - 99 alveus
  - 100 lateral_nucleus
  - 101 parafascicular_nucleus_of_thalamus
  - 102 infundibular_stalk
- P41-16_P1.3
  - 0 background
  - 1 periventricular_white_matter
  - 2 cerebral_cortex
  - 3 white_matter_of_forebrain
  - 4 putamen
  - 5 claustrum
  - 6 body_of_caudate
  - 7 external_segment_of_globus_pallidus
  - 8 substantia_innominata
  - 9 temporal_claustrum
  - 10 stria_terminalis
  - 11 fornix
  - 12 stria_medullaris_of_thalamus
  - 13 internal_segment_of_globus_pallidus
  - 14 optic_tract
  - 15 amygdaloid_complex
  - 16 basolateral_nucleus_(basal_nucleus)
  - 17 ventral_division_of_basomedial_nucleus
  - 18 thalamus
  - 19 anterodorsal_nucleus_of_thalamus
  - 20 anteroventral_nucleus_of_thalamus
  - 21 parataenial_nucleus_of_thalamus
  - 22 parvocellular_division_of_va
  - 23 paraventricular_nucleus
  - 24 zona_incerta
  - 25 amygdalocortical_(corticoamygdaloid)_transition_area
  - 26 inferior_thalamic_peduncle
  - 27 reuniens_nucleus_(medioventral_nucleus)_of_thalamus
  - 28 reticular_nucleus_of_thalamus
  - 29 tuberomammillary_nucleus
  - 30 ventral_medial_nucleus_of_thalamus
  - 31 posterior_hypothalamic_nucleus
  - 32 mammillothalamic_tract
  - 33 medial_subdivision_of_central_nucleus
  - 34 lateral_subdivision_of_central_nucleus
  - 35 magnocellular_(medial)_division_of_md
  - 36 magnocellular_division_of_va
  - 37 central_medial_nucleus_of_thalamus
  - 38 paracentral_nucleus_of_thalamus
  - 39 intermediodorsal__nucleus_of_thalamus
  - 40 choroid_plexus_of_lateral_ventricle
  - 41 amygdalostriatal_transition_area
  - 42 lateral_hypothalamic_area_posterior_part
  - 43 mammillary_peduncle
  - 44 amygdalohippocampal_area
  - 45 central_dorsal_nucleus_of_thalamus
  - 46 supramammillary_nucleus
  - 47 lateral_part_of_medial_mammillary_nucleus
  - 48 medial_part_of_medial_mammillary_nucleus
  - 49 basal_division_of_medial_mammillary_nucleus
  - 50 lateral_mammillary_nucleus
  - 51 lenticular_fasciculus
  - 52 substantia_nigra__reticular_part
  - 53 central_part_of_medial_division_of_md
  - 54 parvocellular_(central)_division_of_md
  - 55 rostral_division_of_vl
  - 56 medial_portion_of_sth
  - 57 laterodorsal_portion_of_sth
  - 58 caudal_subdivision_of_medial_nucleus
  - 59 lateroventral_portion_of_sth
  - 60 thalamic_fasciculus
  - 61 distal_parasubiculum
  - 62 proximal_parasubiculum
  - 63 stratum_lacunosum-moleculare_of_uncal_ca1
  - 64 stratum_radiatum_of_uncal_ca1
  - 65 stratum_pyramidale_of_uncal_ca1
  - 66 stratum_oriens_of_uncal_ca1
  - 67 stratum_lacunosum-moleculare_of_rostral_ca1
  - 68 stratum_radiatum_of_rostral_ca1
  - 69 stratum_pyramidale_of_rostral_ca1
  - 70 stratum_oriens_of_rostral_ca1
  - 71 rostral_subiculum
  - 72 rostral_prosubiculum
  - 73 posteroventral_putamen
  - 74 dorsal_subdivision_of_vlc
  - 75 substantia_nigra__compact_part
  - 76 alveus
  - 77 rostral_presubiculum
  - 78 molecular_layer_of_uncal_dentate_gyrus
  - 79 granular_layer_of_uncal_dentate_gyrus
  - 80 polyform_layer_of_uncal_dentate_gyrus
  - 81 stratum_lacunosum-moleculare_of_uncal_ca2
  - 82 stratum_radiatum_of_uncal_ca2
  - 83 stratum_pyramidale_of_uncal_ca2
  - 84 stratum_oriens_of_uncal_ca2
  - 85 stratum_lacunosum-moleculare_of_uncal_ca3
  - 86 stratum_lucidum__of_uncal_ca3
  - 87 stratum_pyramidale_of_uncal_ca3
  - 88 stratum_oriens_of_uncal_ca3
  - 89 lateral_nucleus
  - 90 lateral_dorsal_nucleus_of_thalamus
  - 91 densocelllular_(paralamellar)_division_of_md
  - 92 multiform_(lateral)_division_of_md
  - 93 ventral_subdivision_of_vlc
  - 94 medial_subdivision_of_vlc_(area_x)
  - 95 body_of_lateral_ventricle
  - 96 midbrain_(mesencephalon)
  - 97 red_nucleus
  - 98 tail_of_caudate
  - 99 rostral_division_of_ventral_posterior_lateral_nucleus
  - 100 parvocellular_division_of_vpm
  - 101 lateral_division_of_centromedian_nucleus_of_thalamus
  - 102 medial_division_of_centromedian_nucleus_of_thalamus
  - 103 parafascicular_nucleus_of_thalamus
  - 104 periventricular_area_of_thalamus
  - 105 nucleus_of_the_field_of_forel
  - 106 parabrachial_pigmented_nucleus
  - 107 molecular_layer_of_rostral_dentate_gyrus
  - 108 granular_layer_of_rostral_dentate_gyrus
  - 109 polyform_layer_of_rostral_dentate_gyrus
  - 110 stratum_lacunosum-moleculare_of_rostral_ca2
  - 111 stratum_radiatum_of_rostral_ca2
  - 112 stratum_pyramidale_of_rostral_ca2
  - 113 stratum_oriens_of_rostral_ca2
  - 114 stratum_lacunosum-moleculare_of_rostral_ca3
  - 115 stratum_lucidum__of_rostral_ca3
  - 116 stratum_pyramidale_of_rostral_ca3
  - 117 stratum_oriens_of_rostral_ca3
  - 118 ventral_posterior_medial_nucleus
  - 119 lateral_division_of_central_lateral_nucleus
  - 120 subparafascicular_nucleus_of_thalamus
  - 121 lateral_habenular_nucleus
  - 122 white_matter_of_midbrain
  - 123 parasubthalamic_nucleus
  - 124 hippocampus_(hippocampal_formation)
  - 125 commissural_nucleus
- P41-16_P2.4
  - 0 background
  - 1 cerebral_cortex
  - 2 white_matter_of_forebrain
  - 3 blood_vessels_of_forebrain
  - 4 putamen
  - 5 claustrum
  - 6 body_of_caudate
  - 7 external_segment_of_globus_pallidus
  - 8 stria_terminalis
  - 9 fornix
  - 10 stria_medullaris_of_thalamus
  - 11 optic_tract
  - 12 thalamus
  - 13 parataenial_nucleus_of_thalamus
  - 14 zona_incerta
  - 15 reticular_nucleus_of_thalamus
  - 16 magnocellular_(medial)_division_of_md
  - 17 choroid_plexus_of_lateral_ventricle
  - 18 substantia_nigra__reticular_part
  - 19 parvocellular_(central)_division_of_md
  - 20 medial_portion_of_sth
  - 21 inferior_horn_of_lateral_ventricle
  - 22 stratum_lacunosum-moleculare_of_rostral_ca1
  - 23 stratum_radiatum_of_rostral_ca1
  - 24 stratum_pyramidale_of_rostral_ca1
  - 25 stratum_oriens_of_rostral_ca1
  - 26 rostral_subiculum
  - 27 dorsal_subdivision_of_vlc
  - 28 substantia_nigra__compact_part
  - 29 alveus
  - 30 stratum_lacunosum-moleculare_of_uncal_ca3
  - 31 stratum_lucidum__of_uncal_ca3
  - 32 stratum_pyramidale_of_uncal_ca3
  - 33 stratum_oriens_of_uncal_ca3
  - 34 lateral_dorsal_nucleus_of_thalamus
  - 35 densocelllular_(paralamellar)_division_of_md
  - 36 multiform_(lateral)_division_of_md
  - 37 midbrain_(mesencephalon)
  - 38 red_nucleus
  - 39 parvocellular_division_of_vpm
  - 40 lateral_division_of_centromedian_nucleus_of_thalamus
  - 41 medial_division_of_centromedian_nucleus_of_thalamus
  - 42 parafascicular_nucleus_of_thalamus
  - 43 periventricular_area_of_thalamus
  - 44 parabrachial_pigmented_nucleus
  - 45 molecular_layer_of_rostral_dentate_gyrus
  - 46 granular_layer_of_rostral_dentate_gyrus
  - 47 polyform_layer_of_rostral_dentate_gyrus
  - 48 stratum_lacunosum-moleculare_of_rostral_ca2
  - 49 stratum_radiatum_of_rostral_ca2
  - 50 stratum_pyramidale_of_rostral_ca2
  - 51 stratum_oriens_of_rostral_ca2
  - 52 stratum_lacunosum-moleculare_of_rostral_ca3
  - 53 stratum_lucidum__of_rostral_ca3
  - 54 stratum_pyramidale_of_rostral_ca3
  - 55 stratum_oriens_of_rostral_ca3
  - 56 ventral_posterior_medial_nucleus
  - 57 ventral_posterior_inferior_nucleus
  - 58 lateral_division_of_central_lateral_nucleus
  - 59 lateral_habenular_nucleus
  - 60 white_matter_of_midbrain
  - 61 lateral_posterior_nucleus_of_thalamus
  - 62 basal_ventral_medial_nucleus
  - 63 dorsal_division_of_central_lateral_nucleus
  - 64 medial_habenular_nucleus
  - 65 anterior_nucleus_of_pulvinar
  - 66 caudal_division_of_ventral_posterior_lateral_nucleus
  - 67 fimbria
  - 68 periaqueductal_gray_substance
  - 69 precommissural_nucleus
  - 70 interstitial_nucleus_of_posterior_commissure
  - 71 subcommissural_organ
  - 72 posterior_commissure
  - 73 cerebral_aqueduct
  - 74 posterior_nucleus_of_thalamus
  - 75 centromedian_nucleus_of_thalamus
  - 76 dorsal_lateral_geniculate_nucleus
  - 77 pretectal_region
  - 78 peripeduncular_nucleus
  - 79 dorsal_medial_geniculate_nucleus
  - 80 superior_colliculus
  - 81 medial_nucleus_of_pulvinar
  - 82 suprageniculate_nucleus_of_thalamus
  - 83 lateral_nucleus_of_pulvinar
  - 84 inferior_nucleus_of_pulvinar
  - 85 internal_medullary_lamina_of_thalamus
  - 86 superficial_pulvinar_nucleus
  - 87 intergeniculate_pulvinar
  - 88 medial_dorsal_thalamic_nucleus
- P41-16_P3.3
  - 0 background
  - 1 cerebral_cortex
  - 2 white_matter_of_forebrain
  - 3 indusium_griseum
  - 4 body_of_caudate
  - 5 stria_terminalis
  - 6 thalamus
  - 7 reticular_nucleus_of_thalamus
  - 8 stratum_lacunosum-moleculare_of_rostral_ca1
  - 9 stratum_radiatum_of_rostral_ca1
  - 10 stratum_pyramidale_of_rostral_ca1
  - 11 stratum_oriens_of_rostral_ca1
  - 12 rostral_subiculum
  - 13 alveus
  - 14 stratum_lacunosum-moleculare_of_uncal_ca3
  - 15 stratum_lucidum__of_uncal_ca3
  - 16 stratum_pyramidale_of_uncal_ca3
  - 17 stratum_oriens_of_uncal_ca3
  - 18 molecular_layer_of_rostral_dentate_gyrus
  - 19 granular_layer_of_rostral_dentate_gyrus
  - 20 polyform_layer_of_rostral_dentate_gyrus
  - 21 stratum_lacunosum-moleculare_of_rostral_ca2
  - 22 stratum_radiatum_of_rostral_ca2
  - 23 stratum_pyramidale_of_rostral_ca2
  - 24 stratum_oriens_of_rostral_ca2
  - 25 stratum_lacunosum-moleculare_of_rostral_ca3
  - 26 stratum_lucidum__of_rostral_ca3
  - 27 stratum_pyramidale_of_rostral_ca3
  - 28 stratum_oriens_of_rostral_ca3
  - 29 lateral_posterior_nucleus_of_thalamus
  - 30 fimbria
  - 31 dorsal_lateral_geniculate_nucleus
  - 32 hippocampus_(hippocampal_formation)
  - 33 medial_nucleus_of_pulvinar
  - 34 hippocampal_commissure
  - 35 crus_of_the_fornix
  - 36 lateral_nucleus_of_pulvinar
  - 37 inferior_nucleus_of_pulvinar
  - 38 brachium_of_superior_colliculus
  - 39 molecular_layer_of_caudal_dentate_gyrus
  - 40 granular_layer_of_caudal_dentate_gyrus
  - 41 polyform_layer_of_caudal_dentate_gyrus
  - 42 stratum_lacunosum-moleculare_of_caudal_ca1
  - 43 stratum_radiatum_of_caudal_ca1
  - 44 stratum_pyramidale_of_caudal_ca1
  - 45 stratum_oriens_of_caudal_ca1
  - 46 stratum_lacunosum-moleculare_of_caudal_ca2
  - 47 stratum_radiatum_of_caudal_ca2
  - 48 stratum_pyramidale_of_caudal_ca2
  - 49 stratum_oriens_of_caudal_ca2
  - 50 stratum_lacunosum-moleculare_of_caudal_ca3
  - 51 stratum_lucidum__of_caudal_ca3
  - 52 stratum_pyramidale_of_caudal_ca3
  - 53 stratum_oriens_of_caudal_ca3
  - 54 caudal_subiculum
  - 55 diffuse_pulvinar_nucleus
- P41-16_P4.1
  - 0 background
  - 1 cerebral_cortex
  - 2 white_matter_of_forebrain
  - 3 indusium_griseum
  - 4 stratum_lacunosum-moleculare_of_rostral_ca1
  - 5 stratum_radiatum_of_rostral_ca1
  - 6 stratum_pyramidale_of_rostral_ca1
  - 7 stratum_oriens_of_rostral_ca1
  - 8 stratum_pyramidale_of_caudal_ca1
  - 9 stratum_pyramidale_of_caudal_ca2
  - 10 stratum_pyramidale_of_caudal_ca3
  - 11 caudal_subiculum
  - 12 caudal_prosubiculum
  - 13 caudal_dentate_gyrus
  - 14 supracallosal_subiculum
- P57-16_A1.3
  - 0 background
  - 1 periventricular_white_matter
  - 2 cerebral_cortex
  - 3 white_matter_of_forebrain
  - 4 peri-caudate_ependymal_and_subependymal_zone
  - 5 putamen
  - 6 anterior_olfactory_nucleus
  - 7 claustrum
  - 8 ventral_division_of_lateral_septal_nucleus
  - 9 basal_nucleus_of_meynert
  - 10 diagonal_band
  - 11 bed_nucleus_of_stria_terminalis
  - 12 body_of_caudate
  - 13 external_segment_of_globus_pallidus
  - 14 ventral_pallidus
  - 15 lateral_olfactory_area
  - 16 substantia_innominata
  - 17 darkly-stained_nanocellular_islands_of_si
  - 18 anterior_commissure
  - 19 lightly-stained_parvocellular_islands_of_si
  - 20 preoptic_region_of_hypothalamus
  - 21 lateral_preoptic_area
  - 22 median_preoptic_nucleus
  - 23 layer_i_of_piriform_cortex
  - 24 layer_ii_of_piriform_cortex
  - 25 layer_iii_of_piriform_cortex
  - 26 nucleus_subputaminalis
  - 27 suprachiasmatic_nucleus
  - 28 optic_chiasm
  - 29 organum_vasculosum_laminae_terminalis
  - 30 temporal_claustrum
  - 31 anteroventral_periventricular_nucleus
  - 32 supraoptic_region_of_hypothalamus
  - 33 uncinate_nucleus
  - 34 stria_terminalis
  - 35 medial_preoptic_nucleus
  - 36 anterior_hypothalamic_nucleus
  - 37 paraventricular_nucleus_of_hypothalamus
  - 38 supraoptic_nucleus
  - 39 fornix
  - 40 stria_medullaris_of_thalamus
  - 41 internal_segment_of_globus_pallidus
  - 42 periventricular_nucleus__supraoptic_portion
  - 43 optic_tract
  - 44 supraoptic_dicussation
  - 45 internal_medullary_lamina_of_globus_pallidus
  - 46 amygdaloid_complex
  - 47 anterior_amygdaloid_area
  - 48 basolateral_nucleus_(basal_nucleus)
  - 49 ventral_division_of_basomedial_nucleus
  - 50 thalamus
  - 51 anterodorsal_nucleus_of_thalamus
  - 52 anteroventral_nucleus_of_thalamus
  - 53 parataenial_nucleus_of_thalamus
  - 54 parvocellular_division_of_va
  - 55 fasciculosus_nucleus_of_thalamus
  - 56 rhomboid_(central)_nucleus_of_thalamus
  - 57 paraventricular_nucleus
  - 58 zona_incerta
  - 59 periventricular_nucleus__tuberal_portion
  - 60 juxtaparaventricular_lateral_hypothalamic_area
  - 61 dorsomedial_hypothalamic_nucleus
  - 62 lateral_hypothalamic_area__tuberal_part
  - 63 accessory_secretory_cells_of_lateral_hypothalamus
  - 64 pallidohypothalamic_area
  - 65 medial_corticohypothalamic_tract
  - 66 ventral_subdivision_of_coa
  - 67 paralaminar_nucleus
  - 68 amygdalocortical_(corticoamygdaloid)_transition_area
  - 69 dorsal_part_of_ventromedial_hypothalamic_nucleus
  - 70 ventral_part_of_ventromedial_hypothalamic_nucleus
  - 71 arcuate_nucleus_of_hypothalamus
  - 72 inferior_thalamic_peduncle
  - 73 anteromedial_nucleus_of_thalamus
  - 74 reuniens_nucleus_(medioventral_nucleus)_of_thalamus
  - 75 reticular_nucleus_of_thalamus
  - 76 central_part_of_ventromedial_hypothalamic_nucleus
  - 77 tuberomammillary_nucleus
  - 78 interthalamic_adhesion_(massa_intermedia)
  - 79 lateral_tuberal_nuclei
  - 80 ventral_medial_nucleus_of_thalamus
  - 81 posterior_hypothalamic_nucleus
  - 82 medial_subdivision_of_central_nucleus
  - 83 lateral_subdivision_of_central_nucleus
  - 84 rostral_subdivision_of_medial_nucleus
  - 85 magnocellular_(medial)_division_of_md
  - 86 magnocellular_division_of_va
  - 87 central_medial_nucleus_of_thalamus
  - 88 paracentral_nucleus_of_thalamus
  - 89 intermediodorsal__nucleus_of_thalamus
  - 90 dorsal_hypothalamic_area
  - 91 amygdalostriatal_transition_area
  - 92 lateral__hypothalamic_area__posterior_part
  - 93 mammillary_peduncle
  - 94 amygdalohippocampal_area
  - 95 central_dorsal_nucleus_of_thalamus
  - 96 supramammillary_nucleus
  - 97 lateral_part_of_medial_mammillary_nucleus
  - 98 medial_part_of_medial_mammillary_nucleus
  - 99 basal_division_of_medial_mammillary_nucleus
  - 100 lateral_mammillary_nucleus
  - 101 lenticular_fasciculus
  - 102 central_part_of_medial_division_of_md
  - 103 parvocellular_(central)_division_of_md
  - 104 rostral_division_of_vl
  - 105 mammillotegmental_tract
  - 106 uncal_subiculum
  - 107 inferior_horn_of_lateral_ventricle
  - 108 stratum_lacunosum-moleculare_of_uncal_ca1
  - 109 stratum_radiatum_of_uncal_ca1
  - 110 stratum_pyramidale_of_uncal_ca1
  - 111 stratum_oriens_of_uncal_ca1
  - 112 stratum_lacunosum-moleculare_of_rostral_ca1
  - 113 stratum_radiatum_of_rostral_ca1
  - 114 stratum_pyramidale_of_rostral_ca1
  - 115 stratum_oriens_of_rostral_ca1
  - 116 rostral_subiculum
  - 117 posteroventral_putamen
  - 118 dorsal_subdivision_of_vlc
  - 119 alveus
  - 120 stratum_lacunosum-moleculare_of_uncal_ca2
  - 121 stratum_radiatum_of_uncal_ca2
  - 122 stratum_pyramidale_of_uncal_ca2
  - 123 stratum_oriens_of_uncal_ca2
  - 124 stratum_lacunosum-moleculare_of_uncal_ca3
  - 125 stratum_lucidum__of_uncal_ca3
  - 126 stratum_pyramidale_of_uncal_ca3
  - 127 stratum_oriens_of_uncal_ca3
  - 128 lateral_nucleus
  - 129 rostral_dentate_gyrus
  - 130 molecular_layer_of_rostral_dentate_gyrus
  - 131 granular_layer_of_rostral_dentate_gyrus
  - 132 polyform_layer_of_rostral_dentate_gyrus
  - 133 stratum_lacunosum-moleculare_of_rostral_ca2
  - 134 stratum_radiatum_of_rostral_ca2
  - 135 stratum_pyramidale_of_rostral_ca2
  - 136 stratum_oriens_of_rostral_ca2
  - 137 stratum_lacunosum-moleculare_of_rostral_ca3
  - 138 stratum_lucidum__of_rostral_ca3
  - 139 stratum_pyramidale_of_rostral_ca3
  - 140 stratum_oriens_of_rostral_ca3
  - 141 infundibular_stalk
  - 142 internal_medullary_lamina_of_thalamus
- P57-16_P2.2
  - 0 background
  - 1 cerebral_cortex
  - 2 white_matter_of_forebrain
  - 3 head_of_caudate
  - 4 blood_vessels_of_forebrain
  - 5 putamen
  - 6 claustrum
  - 7 stria_terminalis
  - 8 fornix
  - 9 stria_medullaris_of_thalamus
  - 10 thalamus
  - 11 zona_incerta
  - 12 reticular_nucleus_of_thalamus
  - 13 magnocellular_(medial)_division_of_md
  - 14 choroid_plexus_of_lateral_ventricle
  - 15 parvocellular_(central)_division_of_md
  - 16 inferior_horn_of_lateral_ventricle
  - 17 stratum_lacunosum-moleculare_of_rostral_ca1
  - 18 stratum_radiatum_of_rostral_ca1
  - 19 stratum_pyramidale_of_rostral_ca1
  - 20 stratum_oriens_of_rostral_ca1
  - 21 rostral_subiculum
  - 22 dorsal_subdivision_of_vlc
  - 23 alveus
  - 24 lateral_dorsal_nucleus_of_thalamus
  - 25 multiform_(lateral)_division_of_md
  - 26 body_of_lateral_ventricle
  - 27 midbrain_(mesencephalon)
  - 28 rostral_dentate_gyrus
  - 29 tail_of_caudate
  - 30 cerebellothalamic_tract
  - 31 molecular_layer_of_rostral_dentate_gyrus
  - 32 granular_layer_of_rostral_dentate_gyrus
  - 33 polyform_layer_of_rostral_dentate_gyrus
  - 34 stratum_lacunosum-moleculare_of_rostral_ca2
  - 35 stratum_radiatum_of_rostral_ca2
  - 36 stratum_pyramidale_of_rostral_ca2
  - 37 stratum_oriens_of_rostral_ca2
  - 38 stratum_lacunosum-moleculare_of_rostral_ca3
  - 39 stratum_lucidum__of_rostral_ca3
  - 40 stratum_pyramidale_of_rostral_ca3
  - 41 stratum_oriens_of_rostral_ca3
  - 42 lateral_division_of_central_lateral_nucleus
  - 43 lateral_habenular_nucleus
  - 44 pyramidal_cells_of_rostral_ca4
  - 45 lateral_posterior_nucleus_of_thalamus
  - 46 dorsal_division_of_central_lateral_nucleus
  - 47 anterior_nucleus_of_pulvinar
  - 48 caudal_division_of_ventral_posterior_lateral_nucleus
  - 49 fimbria
  - 50 periaqueductal_gray_substance
  - 51 interstitial_nucleus_of_posterior_commissure
  - 52 subcommissural_organ
  - 53 posterior_commissure
  - 54 posterior_nucleus_of_thalamus
  - 55 dorsal_lateral_geniculate_nucleus
  - 56 limitans_nucleus
  - 57 peripeduncular_nucleus
  - 58 dorsal_medial_geniculate_nucleus
  - 59 medial_nucleus_of_pulvinar
  - 60 limitans_part_of_medial_geniculate_nucleus
  - 61 lateral_nucleus_of_pulvinar
  - 62 inferior_nucleus_of_pulvinar
  - 63 brachium_of_superior_colliculus
  - 64 internal_medullary_lamina_of_thalamus
  - 65 superficial_pulvinar_nucleus
  - 66 intergeniculate_pulvinar
  - 67 diffuse_pulvinar_nucleus
  - 68 medial_dorsal_thalamic_nucleus
- P57-16_P3.3
  - 0 background
  - 1 cerebral_cortex
  - 2 white_matter_of_forebrain
  - 3 stria_terminalis
  - 4 fornix
  - 5 thalamus
  - 6 choroid_plexus_of_lateral_ventricle
  - 7 stratum_lacunosum-moleculare_of_rostral_ca1
  - 8 stratum_radiatum_of_rostral_ca1
  - 9 stratum_pyramidale_of_rostral_ca1
  - 10 stratum_oriens_of_rostral_ca1
  - 11 rostral_subiculum
  - 12 alveus
  - 13 body_of_lateral_ventricle
  - 14 tail_of_caudate
  - 15 molecular_layer_of_rostral_dentate_gyrus
  - 16 granular_layer_of_rostral_dentate_gyrus
  - 17 polyform_layer_of_rostral_dentate_gyrus
  - 18 stratum_lacunosum-moleculare_of_rostral_ca2
  - 19 stratum_radiatum_of_rostral_ca2
  - 20 stratum_pyramidale_of_rostral_ca2
  - 21 stratum_oriens_of_rostral_ca2
  - 22 stratum_lacunosum-moleculare_of_rostral_ca3
  - 23 stratum_lucidum__of_rostral_ca3
  - 24 stratum_pyramidale_of_rostral_ca3
  - 25 stratum_oriens_of_rostral_ca3
  - 26 pyramidal_cells_of_rostral_ca4
  - 27 fimbria
  - 28 molecular_layer_of_caudal_dentate_gyrus
  - 29 granular_layer_of_caudal_dentate_gyrus
  - 30 polyform_layer_of_caudal_dentate_gyrus
  - 31 stratum_lacunosum-moleculare_of_caudal_ca1
  - 32 stratum_radiatum_of_caudal_ca1
  - 33 stratum_pyramidale_of_caudal_ca1
  - 34 stratum_oriens_of_caudal_ca1
  - 35 stratum_lacunosum-moleculare_of_caudal_ca2
  - 36 stratum_radiatum_of_caudal_ca2
  - 37 stratum_pyramidale_of_caudal_ca2
  - 38 stratum_oriens_of_caudal_ca2
  - 39 stratum_lacunosum-moleculare_of_caudal_ca3
  - 40 stratum_lucidum__of_caudal_ca3
  - 41 stratum_pyramidale_of_caudal_ca3
  - 42 stratum_oriens_of_caudal_ca3
  - 43 pyramidal_cells_of_caudal_ca4
  - 44 caudal_subiculum
  - 45 forceps_inferior
- P58_16_A1.2
  - 0 background
  - 1 cerebral_cortex
  - 2 white_matter_of_forebrain
  - 3 blood_vessels_of_forebrain
  - 4 putamen
  - 5 core_of_nucleus_accumbens
  - 6 claustrum
  - 7 medial_septal_nucleus
  - 8 basal_nucleus_of_meynert
  - 9 bed_nucleus_of_stria_terminalis
  - 10 body_of_caudate
  - 11 external_segment_of_globus_pallidus
  - 12 substantia_innominata
  - 13 anterior_commissure
  - 14 preoptic_region_of_hypothalamus
  - 15 lateral_preoptic_area
  - 16 median_preoptic_nucleus
  - 17 piriform_cortex
  - 18 anteromedial_preoptic_nucleus
  - 19 suprachiasmatic_nucleus
  - 20 optic_chiasm
  - 21 organum_vasculosum_laminae_terminalis
  - 22 medial_forebrain_bundle
  - 23 supraoptic_region_of_hypothalamus
  - 24 medial_preoptic_nucleus
  - 25 anterior_hypothalamic_nucleus
  - 26 paraventricular_nucleus_of_hypothalamus
  - 27 supraoptic_nucleus
  - 28 subparaventricular_zone
  - 29 median_eminence
  - 30 fornix
  - 31 stria_medullaris_of_thalamus
  - 32 internal_segment_of_globus_pallidus
  - 33 optic_tract
  - 34 supraoptic_dicussation
  - 35 amygdaloid_complex
  - 36 anterior_amygdaloid_area
  - 37 ventral_division_of_basomedial_nucleus
  - 38 thalamus
  - 39 anteroventral_nucleus_of_thalamus
  - 40 parvocellular_division_of_va
  - 41 fasciculosus_nucleus_of_thalamus
  - 42 rhomboid_(central)_nucleus_of_thalamus
  - 43 paraventricular_nucleus
  - 44 zona_incerta
  - 45 periventricular_nucleus__tuberal_portion
  - 46 juxtaparaventricular_lateral_hypothalamic_area
  - 47 dorsomedial_hypothalamic_nucleus
  - 48 lateral_hypothalamic_area__tuberal_part
  - 49 pallidohypothalamic_area
  - 50 medial_corticohypothalamic_tract
  - 51 ventral_subdivision_of_coa
  - 52 amygdalocortical_(corticoamygdaloid)_transition_area
  - 53 dorsal_part_of_ventromedial_hypothalamic_nucleus
  - 54 ventral_part_of_ventromedial_hypothalamic_nucleus
  - 55 arcuate_nucleus_of_hypothalamus
  - 56 inferior_thalamic_peduncle
  - 57 anteromedial_nucleus_of_thalamus
  - 58 reuniens_nucleus_(medioventral_nucleus)_of_thalamus
  - 59 reticular_nucleus_of_thalamus
  - 60 central_part_of_ventromedial_hypothalamic_nucleus
  - 61 tuberomammillary_nucleus
  - 62 lateral_tuberal_nuclei
  - 63 magnocellular_nucleus_of_lateral_hypothalamic_area
  - 64 ventral_medial_nucleus_of_thalamus
  - 65 posterior_hypothalamic_nucleus
  - 66 mammillothalamic_tract
  - 67 medial_subdivision_of_central_nucleus
  - 68 magnocellular_(medial)_division_of_md
  - 69 magnocellular_division_of_va
  - 70 central_medial_nucleus_of_thalamus
  - 71 paracentral_nucleus_of_thalamus
  - 72 intermediodorsal__nucleus_of_thalamus
  - 73 dorsal_hypothalamic_area
  - 74 lateral__hypothalamic_area__posterior_part
  - 75 mammillary_peduncle
  - 76 amygdalohippocampal_area
  - 77 supramammillary_nucleus
  - 78 lateral_part_of_medial_mammillary_nucleus
  - 79 medial_part_of_medial_mammillary_nucleus
  - 80 basal_division_of_medial_mammillary_nucleus
  - 81 lateral_mammillary_nucleus
  - 82 parvocellular_(central)_division_of_md
  - 83 caudal_subdivision_of_medial_nucleus
  - 84 infundibular_stalk
- P58_16_A1.3
  - 0 background
  - 1 periventricular_white_matter
  - 2 cerebral_cortex
  - 3 white_matter_of_forebrain
  - 4 piriform_cortex
  - 5 temporal_claustrum
  - 6 amygdaloid_complex
  - 7 anterior_amygdaloid_area
  - 8 basolateral_nucleus_(basal_nucleus)
  - 9 ventral_division_of_basomedial_nucleus
  - 10 paralaminar_nucleus
  - 11 amygdalocortical_(corticoamygdaloid)_transition_area
  - 12 dorsal_subdivision_of_coa
  - 13 medial_subdivision_of_central_nucleus
  - 14 lateral_subdivision_of_central_nucleus
  - 15 amygdalostriatal_transition_area
  - 16 amygdalohippocampal_area
  - 17 amygdalohippocampal_transition_area
  - 18 uncal_subiculum
  - 19 inferior_horn_of_lateral_ventricle
  - 20 distal_parasubiculum
  - 21 stratum_lacunosum-moleculare_of_uncal_ca1
  - 22 stratum_radiatum_of_uncal_ca1
  - 23 stratum_pyramidale_of_uncal_ca1
  - 24 stratum_oriens_of_uncal_ca1
  - 25 stratum_lacunosum-moleculare_of_rostral_ca1
  - 26 stratum_radiatum_of_rostral_ca1
  - 27 stratum_pyramidale_of_rostral_ca1
  - 28 stratum_oriens_of_rostral_ca1
  - 29 rostral_subiculum
  - 30 posteroventral_putamen
  - 31 alveus
  - 32 rostral_presubiculum
  - 33 stratum_lacunosum-moleculare_of_uncal_ca2
  - 34 stratum_radiatum_of_uncal_ca2
  - 35 stratum_pyramidale_of_uncal_ca2
  - 36 stratum_oriens_of_uncal_ca2
  - 37 lateral_nucleus
  - 38 rostral_dentate_gyrus
- P58_16_P1.2
  - 0 background
  - 1 cerebral_cortex
  - 2 white_matter_of_forebrain
  - 3 putamen
  - 4 claustrum
  - 5 body_of_caudate
  - 6 external_segment_of_globus_pallidus
  - 7 substantia_innominata
  - 8 stria_terminalis
  - 9 fornix
  - 10 stria_medullaris_of_thalamus
  - 11 internal_segment_of_globus_pallidus
  - 12 optic_tract
  - 13 amygdaloid_complex
  - 14 thalamus
  - 15 anterodorsal_nucleus_of_thalamus
  - 16 anteroventral_nucleus_of_thalamus
  - 17 parataenial_nucleus_of_thalamus
  - 18 parvocellular_division_of_va
  - 19 paraventricular_nucleus
  - 20 zona_incerta
  - 21 periventricular_nucleus__tuberal_portion
  - 22 amygdalocortical_(corticoamygdaloid)_transition_area
  - 23 reuniens_nucleus_(medioventral_nucleus)_of_thalamus
  - 24 reticular_nucleus_of_thalamus
  - 25 tuberomammillary_nucleus
  - 26 ventral_medial_nucleus_of_thalamus
  - 27 posterior_hypothalamic_nucleus
  - 28 mammillothalamic_tract
  - 29 medial_subdivision_of_central_nucleus
  - 30 magnocellular_(medial)_division_of_md
  - 31 magnocellular_division_of_va
  - 32 central_medial_nucleus_of_thalamus
  - 33 paracentral_nucleus_of_thalamus
  - 34 intermediodorsal__nucleus_of_thalamus
  - 35 anteromedial_large-celled_island_of_md_
  - 36 lateral__hypothalamic_area__posterior_part
  - 37 mammillary_peduncle
  - 38 central_dorsal_nucleus_of_thalamus
  - 39 supramammillary_nucleus
  - 40 lateral_part_of_medial_mammillary_nucleus
  - 41 medial_part_of_medial_mammillary_nucleus
  - 42 basal_division_of_medial_mammillary_nucleus
  - 43 lateral_mammillary_nucleus
  - 44 lenticular_fasciculus
  - 45 substantia_nigra__reticular_part
  - 46 central_part_of_medial_division_of_md
  - 47 parvocellular_(central)_division_of_md
  - 48 rostral_division_of_vl
  - 49 medial_portion_of_sth
  - 50 laterodorsal_portion_of_sth
  - 51 caudal_subdivision_of_medial_nucleus
  - 52 lateroventral_portion_of_sth
  - 53 thalamic_fasciculus
  - 54 dorsal_subdivision_of_vlc
  - 55 substantia_nigra__compact_part
  - 56 lateral_dorsal_nucleus_of_thalamus
  - 57 densocelllular_(paralamellar)_division_of_md
  - 58 multiform_(lateral)_division_of_md
  - 59 ventral_subdivision_of_vlc
  - 60 medial_subdivision_of_vlc_(area_x)
  - 61 midbrain_(mesencephalon)
  - 62 red_nucleus
  - 63 root_of_oculomotor_nerve
  - 64 rostral_division_of_ventral_posterior_lateral_nucleus
  - 65 parvocellular_division_of_vpm
  - 66 lateral_division_of_centromedian_nucleus_of_thalamus
  - 67 medial_division_of_centromedian_nucleus_of_thalamus
  - 68 parafascicular_nucleus_of_thalamus
  - 69 periventricular_area_of_thalamus
  - 70 nucleus_of_the_field_of_forel
  - 71 cerebellothalamic_tract
  - 72 ventral_posterior_medial_nucleus
  - 73 ventral_posterior_inferior_nucleus
  - 74 lateral_division_of_central_lateral_nucleus
  - 75 subparafascicular_nucleus_of_thalamus
  - 76 white_matter_of_midbrain
  - 77 ventral_tegmental_area
- P58-16_P1.3
  - 0 background
  - 1 periventricular_white_matter
  - 2 cerebral_cortex
  - 3 white_matter_of_forebrain
  - 4 blood_vessels_of_forebrain
  - 5 claustrum
  - 6 external_segment_of_globus_pallidus
  - 7 stria_terminalis
  - 8 basolateral_nucleus_(basal_nucleus)
  - 9 ventral_division_of_basomedial_nucleus
  - 10 amygdalocortical_(corticoamygdaloid)_transition_area
  - 11 medial_subdivision_of_central_nucleus
  - 12 lateral_subdivision_of_central_nucleus
  - 13 rostral_subdivision_of_medial_nucleus
  - 14 choroid_plexus_of_lateral_ventricle
  - 15 amygdalostriatal_transition_area
  - 16 amygdalohippocampal_area
  - 17 caudal_subdivision_of_medial_nucleus
  - 18 inferior_horn_of_lateral_ventricle
  - 19 stratum_lacunosum-moleculare_of_uncal_ca1
  - 20 stratum_radiatum_of_uncal_ca1
  - 21 stratum_pyramidale_of_uncal_ca1
  - 22 stratum_oriens_of_uncal_ca1
  - 23 stratum_lacunosum-moleculare_of_rostral_ca1
  - 24 stratum_radiatum_of_rostral_ca1
  - 25 stratum_pyramidale_of_rostral_ca1
  - 26 stratum_oriens_of_rostral_ca1
  - 27 rostral_subiculum
  - 28 posteroventral_putamen
  - 29 alveus
  - 30 molecular_layer_of_uncal_dentate_gyrus
  - 31 granular_layer_of_uncal_dentate_gyrus
  - 32 polyform_layer_of_uncal_dentate_gyrus
  - 33 stratum_lacunosum-moleculare_of_uncal_ca2
  - 34 stratum_radiatum_of_uncal_ca2
  - 35 stratum_pyramidale_of_uncal_ca2
  - 36 stratum_oriens_of_uncal_ca2
  - 37 stratum_lacunosum-moleculare_of_uncal_ca3
  - 38 stratum_lucidum__of_uncal_ca3
  - 39 stratum_pyramidale_of_uncal_ca3
  - 40 stratum_oriens_of_uncal_ca3
  - 41 rostral_dentate_gyrus
  - 42 tail_of_caudate
  - 43 molecular_layer_of_rostral_dentate_gyrus
  - 44 granular_layer_of_rostral_dentate_gyrus
  - 45 polyform_layer_of_rostral_dentate_gyrus
  - 46 stratum_lacunosum-moleculare_of_rostral_ca2
  - 47 stratum_radiatum_of_rostral_ca2
  - 48 stratum_pyramidale_of_rostral_ca2
  - 49 stratum_oriens_of_rostral_ca2
  - 50 stratum_lacunosum-moleculare_of_rostral_ca3
  - 51 stratum_lucidum__of_rostral_ca3
  - 52 stratum_pyramidale_of_rostral_ca3
  - 53 stratum_oriens_of_rostral_ca3
  - 54 pyramidal_cells_of_uncal_ca4
  - 55 pyramidal_cells_of_rostral_ca4
- P58-16_P2.2
  - 0 background
  - 1 cerebral_cortex
  - 2 white_matter_of_forebrain
  - 3 putamen
  - 4 body_of_caudate
  - 5 stria_medullaris_of_thalamus
  - 6 thalamus
  - 7 parataenial_nucleus_of_thalamus
  - 8 paraventricular_nucleus
  - 9 zona_incerta
  - 10 reuniens_nucleus_(medioventral_nucleus)_of_thalamus
  - 11 reticular_nucleus_of_thalamus
  - 12 magnocellular_(medial)_division_of_md
  - 13 choroid_plexus_of_lateral_ventricle
  - 14 substantia_nigra__reticular_part
  - 15 parvocellular_(central)_division_of_md
  - 16 dorsal_subdivision_of_vlc
  - 17 substantia_nigra__compact_part
  - 18 lateral_dorsal_nucleus_of_thalamus
  - 19 multiform_(lateral)_division_of_md
  - 20 midbrain_(mesencephalon)
  - 21 red_nucleus
  - 22 parvocellular_division_of_vpm
  - 23 lateral_division_of_centromedian_nucleus_of_thalamus
  - 24 parafascicular_nucleus_of_thalamus
  - 25 parabrachial_pigmented_nucleus
  - 26 cerebellothalamic_tract
  - 27 fasciculus_retroflexus_(habenuno-interpeduncular_tract)
  - 28 ventral_posterior_medial_nucleus
  - 29 ventral_posterior_inferior_nucleus
  - 30 lateral_division_of_central_lateral_nucleus
  - 31 subparafascicular_nucleus_of_thalamus
  - 32 white_matter_of_midbrain
  - 33 lateral_posterior_nucleus_of_thalamus
  - 34 basal_ventral_medial_nucleus
  - 35 dorsal_division_of_central_lateral_nucleus
  - 36 anterior_nucleus_of_pulvinar
  - 37 caudal_division_of_ventral_posterior_lateral_nucleus
  - 38 periaqueductal_gray_substance
  - 39 precommissural_nucleus
  - 40 interstitial_nucleus_of_posterior_commissure
  - 41 posterior_commissure
  - 42 posterior_nucleus_of_thalamus
  - 43 centromedian_nucleus_of_thalamus
  - 44 dorsal_lateral_geniculate_nucleus
  - 45 limitans_nucleus
  - 46 pretectal_region
  - 47 peripeduncular_nucleus
  - 48 medial_lemniscus
  - 49 dorsal_medial_geniculate_nucleus
  - 50 superior_colliculus
  - 51 medial_nucleus_of_pulvinar
  - 52 suprageniculate_nucleus_of_thalamus
  - 53 brachium_of_inferior_colliculus
  - 54 pulvinar_of_thalamus
  - 55 commissural_nucleus
  - 56 superficial_pulvinar_nucleus
  - 57 medial_dorsal_thalamic_nucleus
  - 58 dorsal_superficial_nucleus
  - 59 habenula
  - 60 fascicles_retroflux
- P58-16_P2.3
  - 0 background
  - 1 cerebral_cortex
  - 2 white_matter_of_forebrain
  - 3 body_of_caudate
  - 4 stria_terminalis
  - 5 thalamus
  - 6 choroid_plexus_of_lateral_ventricle
  - 7 stratum_lacunosum-moleculare_of_rostral_ca1
  - 8 stratum_radiatum_of_rostral_ca1
  - 9 stratum_pyramidale_of_rostral_ca1
  - 10 stratum_oriens_of_rostral_ca1
  - 11 rostral_subiculum
  - 12 alveus
  - 13 molecular_layer_of_rostral_dentate_gyrus
  - 14 granular_layer_of_rostral_dentate_gyrus
  - 15 polyform_layer_of_rostral_dentate_gyrus
  - 16 stratum_lacunosum-moleculare_of_rostral_ca2
  - 17 stratum_radiatum_of_rostral_ca2
  - 18 stratum_pyramidale_of_rostral_ca2
  - 19 stratum_oriens_of_rostral_ca2
  - 20 stratum_lacunosum-moleculare_of_rostral_ca3
  - 21 stratum_lucidum__of_rostral_ca3
  - 22 stratum_pyramidale_of_rostral_ca3
  - 23 stratum_oriens_of_rostral_ca3
  - 24 pyramidal_cells_of_rostral_ca4
  - 25 fimbria
- P58-16_P3.2
  - 0 background
  - 1 middle_frontal_gyrus
  - 2 cerebral_cortex
  - 3 white_matter_of_forebrain
  - 4 indusium_griseum
  - 5 putamen
  - 6 body_of_caudate
  - 7 stria_terminalis
  - 8 thalamus
  - 9 reticular_nucleus_of_thalamus
  - 10 stratum_lacunosum-moleculare_of_rostral_ca1
  - 11 stratum_radiatum_of_rostral_ca1
  - 12 stratum_pyramidale_of_rostral_ca1
  - 13 stratum_oriens_of_rostral_ca1
  - 14 rostral_subiculum
  - 15 alveus
  - 16 molecular_layer_of_rostral_dentate_gyrus
  - 17 granular_layer_of_rostral_dentate_gyrus
  - 18 polyform_layer_of_rostral_dentate_gyrus
  - 19 stratum_lacunosum-moleculare_of_rostral_ca2
  - 20 stratum_radiatum_of_rostral_ca2
  - 21 stratum_pyramidale_of_rostral_ca2
  - 22 stratum_oriens_of_rostral_ca2
  - 23 stratum_lacunosum-moleculare_of_rostral_ca3
  - 24 stratum_lucidum__of_rostral_ca3
  - 25 stratum_pyramidale_of_rostral_ca3
  - 26 stratum_oriens_of_rostral_ca3
  - 27 white_matter_of_midbrain
  - 28 pyramidal_cells_of_rostral_ca4
  - 29 lateral_posterior_nucleus_of_thalamus
  - 30 fimbria
  - 31 periaqueductal_gray_substance
  - 32 pretectal_region
  - 33 superior_colliculus
  - 34 medial_nucleus_of_pulvinar
  - 35 lateral_nucleus_of_pulvinar
  - 36 inferior_nucleus_of_pulvinar
  - 37 molecular_layer_of_caudal_dentate_gyrus
  - 38 granular_layer_of_caudal_dentate_gyrus
  - 39 polyform_layer_of_caudal_dentate_gyrus
  - 40 stratum_lacunosum-moleculare_of_caudal_ca1
  - 41 stratum_radiatum_of_caudal_ca1
  - 42 stratum_pyramidale_of_caudal_ca1
  - 43 stratum_oriens_of_caudal_ca1
  - 44 stratum_lacunosum-moleculare_of_caudal_ca2
  - 45 stratum_radiatum_of_caudal_ca2
  - 46 stratum_pyramidale_of_caudal_ca2
  - 47 stratum_oriens_of_caudal_ca2
  - 48 stratum_lacunosum-moleculare_of_caudal_ca3
  - 49 stratum_lucidum__of_caudal_ca3
  - 50 stratum_pyramidale_of_caudal_ca3
  - 51 stratum_oriens_of_caudal_ca3
  - 52 pyramidal_cells_of_caudal_ca4
  - 53 caudal_subiculum
  - 54 pulvinar_of_thalamus
  - 55 supracallosal_subiculum
  - 56 commissural_nucleus
  - 57 diffuse_pulvinar_nucleus
