# Supplementary material for: Deep active learning for suggestive segmentation of biomedical image stacks via optimisation of Dice scores and traced boundary length
Source: Med Image Anal. Author manuscript; Available in PMC 2024 Nov 29. (PMC11605667; doi:10.1016/j.media.2022.102549)
Supplement: supplementary figures [file NIHMS2036326-supplement-supplementary_figures.docx]

***Figure S1***. Qualitative results for a synthetically deformed MRI stack comparing the proposed framework with three commonly used baselines: entropy based (H), random, and uniform. The figure represents a snapshot of the manually annotated ROIs when the different methods reach a Dice score of 90%. From top to bottom the rows show the ground truth labels which have been synthetically created, the initial training set consisting of manual annotation of one instance for each structure in the image stack, the proposed framework and the competing baselines. In parenthesis we report the corresponding manual effort (in % of the total effort). The label B indicates the background has been annotated; we omit this for the ground truth and the slice-wise uniform case as they require whole slice annotation.

***Figure S2***. Qualitative results for a histology block comparing the proposed framework with three commonly used baselines: entropy based (H), random, and slice-wise uniform. The figure represents a snapshot of the manually annotated ROIs when the different methods reach a Dice score of 90%. From top to bottom the rows show the ground truth labels, the initial training set consisting of manual annotation of one instance for each structure in the block, the proposed framework and the competing baselines. In parenthesis we report the corresponding manual effort (in % of the total effort). The label B indicates the background has been annotated; we omit this for the ground truth and the slice-wise uniform case as they require whole slice annotation.

***Figure S3.*** (**a**, **b**) Brain hemisphere from medial (**a**) and lateral (**b**) views; (**c**) naming convention for anterior (A[1-6]) and posterior coronal slices (P[1-8]) cut from the cerebrum; (**d**) cut slices; (**e**) Blocking of slices; the number of blocks varies across slices, depending on size and shape.

The figure is adapted from *Mancini, M., Casamitjana, A., Peter, L. et al. A multimodal computational pipeline for 3D histology of the human brain. Sci Rep 10, 13839 (2020)*, under the Creative Commons Attribution 4.0 International License.

***Figure S4.*** Size in pixels of the selected ROI at every iteration for the different competing algorithms for a sample MRI volume. Methods relying on entropy tend to choose larger ROIs earlier. Note that the Uniform method requires the labelling of a full slice at each iteration and therefore is not included in this figure.

***Figure S5.*** Size in pixels of the selected ROI at every iteration for the different competing algorithms for a sample histology block (P41-16_P1.3). Methods relying on entropy tend to choose larger ROIs earlier. Note that the Uniform method requires the labelling of a full slice at each iteration and therefore is not included in this figure.
